# GDPLichi: a DNA Damage Repair-Related Gene Classifier for Predicting Lung Adenocarcinoma Immune Checkpoint Inhibitors Response

**DOI:** 10.3389/fonc.2021.733533

**Published:** 2021-12-02

**Authors:** Yang Leng, Shiying Dang, Fei Yin, Tianshun Gao, Xing Xiao, Yi Zhang, Lin Chen, Changfei Qin, Nannan Lai, Xiao-Yong Zhan, Ke Huang, Chuanming Luo, Yang Kang, Nan Wang, Yun Li, Yuhong Liang, Bihui Huang

**Affiliations:** ^1^ Scientific Research Center, The Seventh Affiliated Hospital, Sun Yat-sen University, Shenzhen, China; ^2^ Department of Thoracic Surgery, The Seventh Affiliated Hospital, Sun Yat-sen University, Shenzhen, China; ^3^ Center for Clinical Neuroscience, The Seventh Affiliated Hospital, Sun Yat-sen University, Shenzhen, China; ^4^ School of Medicine, Southern University Of Science And Technology, Shenzhen, China

**Keywords:** DNA damage repair (DDR), immune check inhibitor (ICI), GDPLichi, lung adenocarcinoma, gene classifier

## Abstract

Lung cancer is one of the most common and mortal malignancies, usually with a poor prognosis in its advanced or recurrent stages. Recently, immune checkpoint inhibitors (ICIs) immunotherapy has revolutionized the treatment of human cancers including lung adenocarcinoma (LUAD), and significantly improved patients’ prognoses. However, the prognostic and predictive outcomes differ because of tumor heterogeneity. Here, we present an effective method, GDPLichi (Genes of DNA damage repair to predict LUAD immune checkpoint inhibitors response), as the signature to predict the LUAD patient’s response to the ICIs. GDPLichi utilized only 7 maker genes from 8 DDR pathways to construct the predictive model and classified LUAD patients into two subgroups: low- and high-risk groups. The high-risk group was featured by worse prognosis and decreased B cells, CD8^+^ T cells, CD8^+^ central memory T cells, hematopoietic stem cells (HSC), myeloid dendritic cells (MDC), and immune scores as compared to the low-risk group. However, our research also suggests that the high-risk group was more sensitive to ICIs, which might be explained by increased TMB, neoantigen, immune checkpoint molecules, and immune suppression genes’ expression, but lower TIDE score as compared to the low-risk group. This conclusion was verified in three other LUAD cohort datasets (GSE30219, GSE31210, GSE50081).

## Introduction

Lung cancer ranks the second in incidence and top in mortality among malignancies worldwide (1), of which lung adenocarcinoma is the most common subtype (2). The prognosis of advanced and recurrent lung cancer is usually poor because most standard treatments by cytotoxic anticancer drugs only have limited therapeutic effects. In recent years, with a better understanding of immune response regulation, the immune checkpoint inhibitor (ICI) therapy showed improved survival rates in multiple cancers including non-small-cell lung cancer (NSCLC). The principle of ICIs is to reactivate immune cells by using specific antibodies against inhibitory signaling molecules such as CTLA-4 and PD-1 expressed on tumor and immune cells. Currently, the approved drugs of anti-CTLA-4 (ipilimumab), anti-PD-1 (nivolumab and pembrolizumab), anti-PD-L1 (atezolizumab, avelumab, and durvalumab), and their combinations have performed significant improvements in treating advanced NSCLC patients (3–6). Lung adenocarcinoma accounts for 80% of NSCLC and benefits most from ICIs therapy. However, it was also reported that there were still only partial LUAD patients responsive to ICIs (7)

PD-L1 expression has been widely used as an ICI response predictive marker, but the sensitivity and specificity are not very consistent due to different antibodies and cutoff values used for PD-L1 test (3, 8, 9). Meanwhile, PD-L1 expression cannot accurately reflect the complicated tumor immune microenvironment (10). Recent studies have also reported that tumor mutation burden (TMB) is closely related to the efficacy of ICIs response (11, 12) and can also be used as a predictive marker for the efficacy of ICI treatment. Like PD-L1, the cut-off value of TMB is controversial (13–15). Additionally, TMB alone does not directly produce neoantigen processing by major histocompatibility complex (MHC) class molecules, thus the accuracy of TMB as a predictor for ICI treatment is modest. Neoantigen expressed on tumor cells is one of the main targets for an effective antitumor T-cell response (16), but difficult to be identified. Therefore, identifying novel markers that can efficiently and accurately predict ICI responses is urgent. One promising area for this research is DNA damage repair (DDR). To ensure the integrity of the genome, cells activate DNA damage repair pathways to repair genetic lesions (SNP, Indel, etc.) during the process of DNA replication. DDR consists of eight pathways including miss match repair (MMR), base excision repair (BER), nucleotide excision repair (NER), direct damage repair (DR), homologous dependent recombination (HDR), nonhomologous end joining (NHEJ), fanconi anemia pathway (FA), and translesion DNA synthesis (TLS). Defects in DDR pathways lead to the accumulation of genomic aberrations and an elevated TMB (17–19), thus promoting tumor development (20). Many studies have shown that mutations in DDR pathway genes are associated with ICIs responses (20, 21). Patients who have DDR genomic alterations usually have a better clinical benefit after ICIs therapy (19, 21).

The Tumor Immune Dysfunction and Exclusion (TIDE) algorithm is a computational method that uses gene expression profiles to predict the ICIs response, particularly successful in NSCLC and melanoma (22). TIDE uses a specific set of marker genes to estimate dysfunction of tumor-infiltrating cytotoxic T lymphocyte and exclusion of CTL by an immunosuppressive factor to predict patients’ response to ICIs. Patients with lower TIDE scores have a lower chance of antitumor immune escape, thus having a higher response rate of ICIs treatment (22). The TIDE score exhibited a higher accuracy than PD-L1 expression level and TMB in predicting the overall survival of patients treated with ICIs (20, 23, 24). Some studies also have reported its utility in predicting or evaluating the ICIs efficacy (24–28).

We identified seven significant genes (e.g., DUT, MGMT, POLH, RAD1, RAD17, TYMS, and YWHAG) strongly associated with prognosis from DDR pathways using Cox regression analysis.

Patients with lower expression of DUT, TYMS, and YWHAG but higher expression of MGMT, POLH, RAD1, and RAD17 had a better prognosis. Based on the expressions and weights calculated by Cox regression on these genes, we developed a classifier, GDPLichi (Genes of DDR to Predict LUAD immune checkpoint inhibitors), as the signature to predict the ICIs response. LUAD patients were classified into low- and high-risk groups based on the cutoff of the GDPLichi score. Many features, including PDCD1, CTLA4, PD-L1 expression, TMB, and neoantigen, displayed strong discerning abilities in the survival analysis of these two subgroups. Especially, the high-risk subgroup had a worse prognosis but is presumably more efficacious towards ICIs treatment.

## Materials and Method

### Data Source

**Figure 1:**
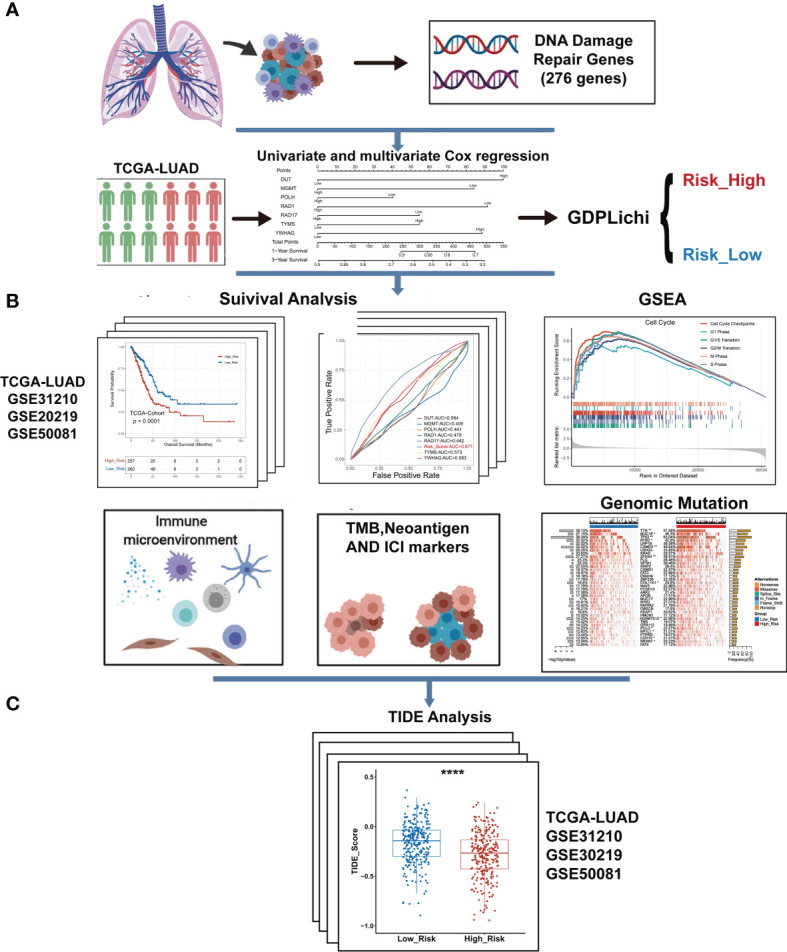
The overall workflow of GDPLichi. **(A)** GDPLichi was constructed by DNA damage-related genes and divided LUAD patients into two subgroups (low- and high-risk). **(B)** GDPLichi can be used for the analyses of survival, GSEA, immune microenvironment, TMB, Neoantigen, immune checkpoint genes (PD-L1, PD-1, CTLA4), and genomic mutation between low- and high-risk subgroups. **(C)** The TIDE algorithm was used to predict the sensitivity to immune checkpoint inhibitors (ICIs) between low- and high-risk groups in four cohorts.

To predict the LUAD ICIs response, we built a multi-step approach called GDPLichi described below (**Figure 1** and **Supplementary Figure S1**). The transcriptome gene expression, genomic data, and clinical phenotype data of 526 TCGA-LUAD samples were downloaded from the website xenabrowser (https://xenabrowser.net/datapages/). TCGA raw RNA-Seq transcriptome count data including 526 LUAD samples were further transferred into a transcript per kilobase mullion (TPM). Three validation groups of raw data, including 438 LUAD samples from 3 cohorts [GSE31210 (29), GSE30219 (30), and GSE50081 (31)], were downloaded from Gene Expression Omnibus (GEO) repository. Then raw data were transferred to expression data using the “Oligo” package in R software. For genes with multiple probes, their expression levels were calculated as the maximum expression level of these probes. Finally, all expression data were normalized and converted to Z-scores.

### GDPLichi Score

First, a univariate Cox regression model was used to assess the association of 276 DNA damage repair-related genes (**Supplementary File 1**) with the overall survival in the TCGA LUAD cohort. P-value was used to identify key genes and genes with P-value < 0.05 were considered as predictive genes (**Supplementary File 2**). Then, 63 predictive genes were selected for multivariate Cox regression and genes with P-value < 0.05 were considered as risk genes (**Supplementary File 3**). Finally, seven risk genes were obtained by multivariate Cox regression and combined to construct the GDPLichi classifier. By combining the expression values of risk genes and weighting by multivariate Cox regression coefficients, the GDPLichi score for each patient was calculated as follows:


$$\text{GDPLichi score}=\Sigma_{i=1}^{n}\;expre_{i}\beta_{i}$$

Here n is the number of prognostic genes, expre_i_ meant the expression value of gene i, and β_i_ represented the regression coefficient of gene i in the multivariate Cox regression analysis. Using the median GDPLichi score as a cutoff value, TCGA and GEO LUAD patients could be classified into low and high-risk groups.

### Gene Set Enrich Analysis, Survival Analysis, Principal Components Analysis, Tumor Microenvironment Analysis, and TIDE

R language 4.0 was applied in this study for the statistical analyses. GSEA was used to explore the pathway enrichment between low- and high-risk groups using the R package “clusterProfiler” (32) on the Reactome pathway database (33) with default parameters. The fold change of gene expressions between two groups was used to rank the genes. The absolute values of the normalized enrichment score (NES) >1 and P-value ≤0.05 were used to screen out significantly enriched pathways. The “survivalROC” package was used to plot the survival ROC curve. The cutoff of survival time was set to 36 months. “Forest plot” was used in the “forestmodel” package and the “factor_separate_line” parameters were set as TRUE. Survival analysis of two groups was carried out by the R package “survminer”. PCA was used by the R packages “FactoMineR” and “factoextra” with the values of all genes’ expression as the input. We used the “xCell” package (34) to estimate relative subsets of immune cells. TIDE Score was calculated with the TIDE algorithm (22) from the website (http://tide.dfci.harvard.edu). All R package parameters can be found in the source analysis code in main_code.R (**Supplementary File 6**).

### Patient Sample Collection

From the TCGA LUAD cohort downloaded from the xenabrowser website, samples with survival, and genomic data were collected. In datasets GSE31210, GSE50081, and GSE30219, lung squamous cell carcinoma samples could be excluded and lung adenocarcinoma with survival data were collected.

## Result

### Construction of GDPLichi

**Figure 2:**
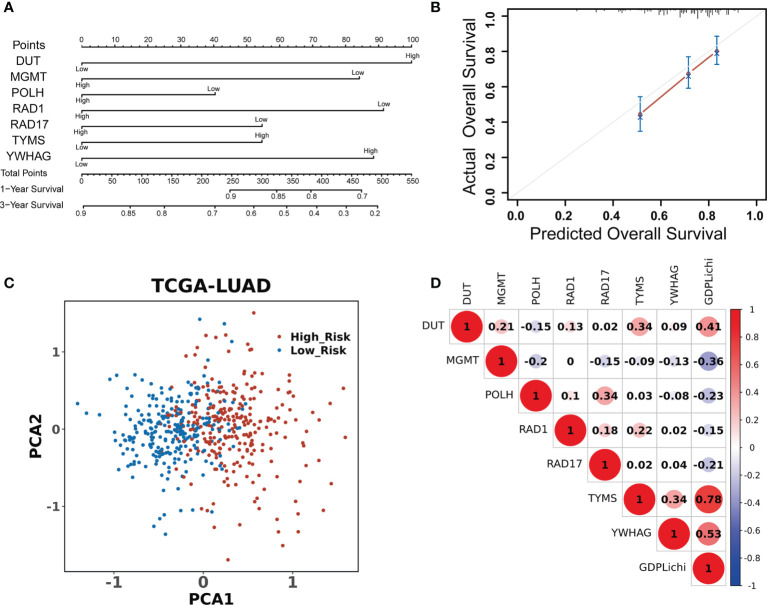
DDR signature accurately predicts the prognosis of LUAD patients. **(A)** Univariate and multivariate Cox regression analyses screened out seven risk genes. The point number represents a score for the relation between the expression of each selected gene and the predicted survival calculated by Cox regression. High and low represent the highest and lowest expression levels of the gene, respectively. Total points were the sum of the individual points from the seven selected genes. Based on the total points, 1-year and 3-year predicted overall survival rates of each LUAD patient were calculated. The higher the number, the lower the predicted survival. **(B)** Multivariate Cox regression analysis of the seven risk genes in predicting the survival of LUAD patients. **(C)** PCA based on the expression profile of the seven risk genes from different risk groups. **(D)** Correlation between the GDPLichi and the seven risk genes in the TCGA LUAD cohort.

A univariate Cox regression model was used to assess the association of 276 DNA damage repair genes with the overall survival in the TCGA LUAD cohort. There were 63 predictive genes screened out with an initial significance (P <0.05). By using these 63 predictive genes as input for multivariate Cox regression, seven risk genes (DUT, MGMT, POLH, RAD1, RAD17, TYMS, and YWHAG) were screened out (**Figure 2A**) and Kaplan–Meier analysis further confirmed the prognostic value of the individual genes (**Suppelementary Figure S2**). The multivariate Cox regression analysis of the above seven risk genes showed high accuracy in predicting the survival of LUAD patients (**Figure 2B**). By combining the expression values of seven risk genes and weighted by COX regression coefficients, the GDPLichi score for each patient was calculated (Described in 2.2). To further facilitate the application of the GDPLichi, the patients were divided into low- and high-risk groups according to the median value of the GDPLichi score. PCA showed that patients could be distinctively clustered according to the selected signatures (the seven risk genes) in the TCGA LUAD cohort (**Figure 2C**) and three GEO validation cohorts (**Suppelementary Figure S3A**). In addition, Spearman’s correlation test indicated that GDPLichi was significantly correlated with the selected genes in the TCGA LUAD cohort (**Figure 2D**) and three GEO validation cohorts (**Figure S3B**).

### Identification of LUAD Subgroups With Prognostic Significance According to GDPLichi

**Figure 3:**
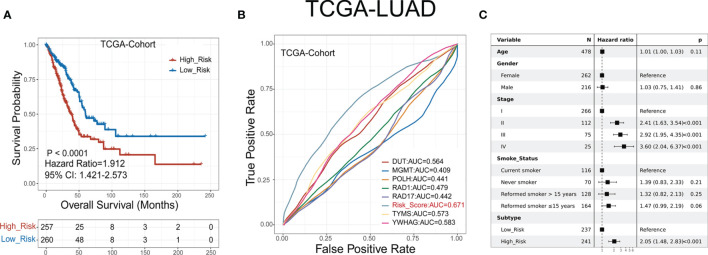
GDPLichi score can function as a prognostic index for LUAD patients. **(A)** Kaplan-Meier plots of the survival probability for low- and high-risk subgroups of TCGA cohort, respectively. **(B)** ROC curves for the performance of the GDPLichi score as well as the seven risk genes of the classifier in TCGA in predicting prognosis. **(C)** Forest plot representation of multivariate Cox model depicting the association between overall survival and LUAD subgroups with other clinical factors considered in the TCGA cohort.

LUAD patients were classified into low- or high-risk groups based on the median GDPLichi score described above. The overall survival analysis for these two subgroups showed a significant difference in the TCGA cohort (**Figure 3A**, P<0.0001) and three GEO validation cohorts (**Suppelementary Figure S4A**).

The hazard ratio of the two subgroups in the TCGA cohort is 1.912 (GSE30219: 2.99, GSE31210: 3.79, GSE50081: 2.43). The 95% confidence interval of two subgroups of TCGA cohort is 1.421-2.573 (GSE30219: 1.585-5.641, GSE31210: 1.72-8.351, GSE50081: 1.356-4.356). The difference remained statistically significant after adjusting for age, gender, stage, and smoking status in the TCGA cohort (**Figure 3C**) and three GEO validation cohorts (**Suppelementary Figure S4C**). To test the practicality of the GDPLichi classifier, we applied ROC (Receiver Operating Characteristic) analyses to the TCGA cohort and found that the GDPLichi score could function as a better prognostic index than any risk gene alone (**Figure 3B**). This result was also validated in the three GEO cohorts (**Suppelementary Figure S4B**). Therefore, the GDPLichi could be a good model to predict the prognosis of LUAD patients.

### GSEA Explored the Pathway Enrichment Between Low- and High-Risk Groups

**Figure 4:**
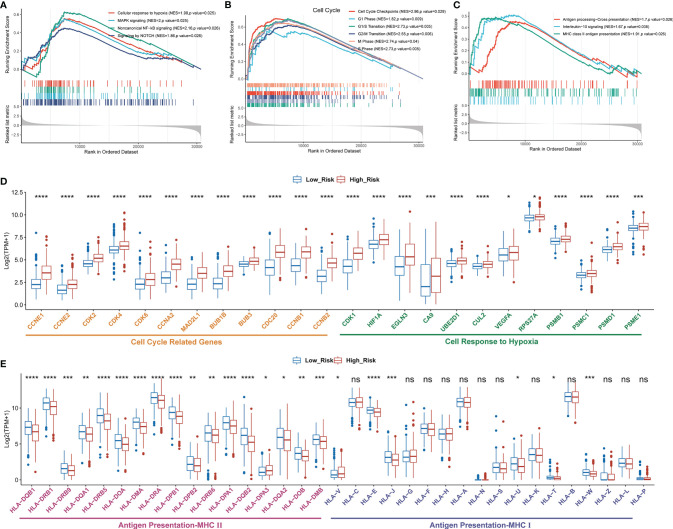
Enriched proliferation-related and cell cycle pathways, but reduced immune-related pathways in the high- as compared to the low-risk group classified by GDPLichi. GSEA plots of proliferation-related pathways **(A)**, cell cycle, **(B)**, and immune-related pathways **(C)**. All transcripts are ranked by the fold change between low- and high-risk subgroups in the TCGA-LUAD cohort. **(D, E)** The difference in the expression of cell cycle-related, cell response to hypoxia, and antigen presentation genes between low and high-risk subgroups. *P < 0.05; **P < 0.01; ***P < 0.001; ****P < 0.0001; ns, no significant difference.

To further investigate the difference of biological mechanisms between low- and high-risk groups divided by GDPLichi, we performed GSEA on the TCGA LUAD cohort. It revealed that cell proliferation-related pathways such as cellular response to hypoxia, MAPK signaling, and noncanonical NF-κB signaling were significantly enriched in the high-risk group (**Figures 4, B**). Meanwhile, cell cycle pathways were also significantly enriched in the high-risk group (**Figures 4B, D**). The results also showed that immune-related pathways such as antigen procession, cross-presentation, interleukin-10 signaling, and MHC class II antigen presentation were significantly enriched (**Figure 4C**). By examining the expression of HLA genes, it was revealed that the expression of MHC II genes in the low-risk group was significantly higher than in the high-risk group (**Figure 4E**). MHC II genes are only expressed in antigen-presenting cells. This may indicate a higher tumor-infiltrating lymphocyte (TIL) in the low-risk group, and ultimately a poorer prognosis.

### The Difference in Tumor Immune Microenvironment Between Low- and High-Risk Groups

**Figure 5:**
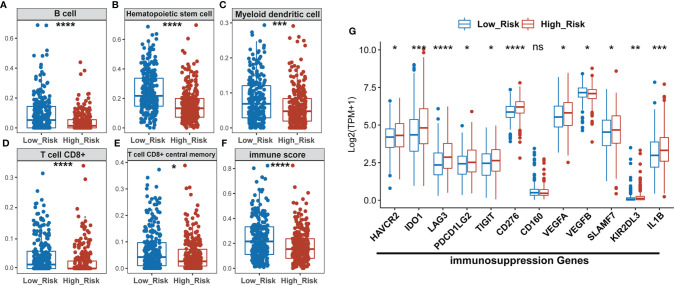
The high-risk LUAD group exhibits relatively lower infiltration of B, HSC, MDC, CD8^+^ T, and memory T cells, and lower immune scores than the low-risk group, but has higher expression of immuno-suppression genes. **(A–F)** Comparison of infiltrating immune cells (xCell) between low- and high-risk groups using xCell algorism. **(G)** Statistical analysis of the expression of immunosuppression genes between low- and high-risk groups. *P < 0.05; **P < 0.01; ***P < 0.001; ****P < 0.0001; ns, no significant difference.

The “xCell” algorithm was employed to estimate the immune cells in malignant tumor tissues between two subgroups using RNA sequencing data. Our results showed that the immune scores, B cells, hematopoietic stem cells (HSC), myeloid dendritic cells (MDC), CD8^+^ T cells, and CD8^+^ central memory T cells were significantly higher in low-risk groups compared to high-risk groups, suggesting a higher TIL in the low-risk group (**Figures 5A–F**). CD8^+^ T cells, also named cytotoxic T cells, are one of the major tumor killer cells and CD8^+^ cell exclusion is strongly associated with tumor immune escape. Therefore, we examined the expression of several immune-suppression genes such as TIM-3 (HAVCR2), IDO1, LAG3, PD-L2 (PDCD1LG2), TIGIT, CD276, CD160, VEGFA, VEGFB, SLAMF7, KIR2DL3, and IL1B between low- and high-risk groups. As shown in **Figure 5G**, the high-risk group had a higher expression of immunosuppression genes than the low-risk group, which might account for higher sensitivity to ICIs in the high-risk subgroup of LUAD patients.

### The Difference in TMB, Neoantigen, and ICIs-Target Expression Between Low- and High-Risk Subgroups

**Figure 6:**
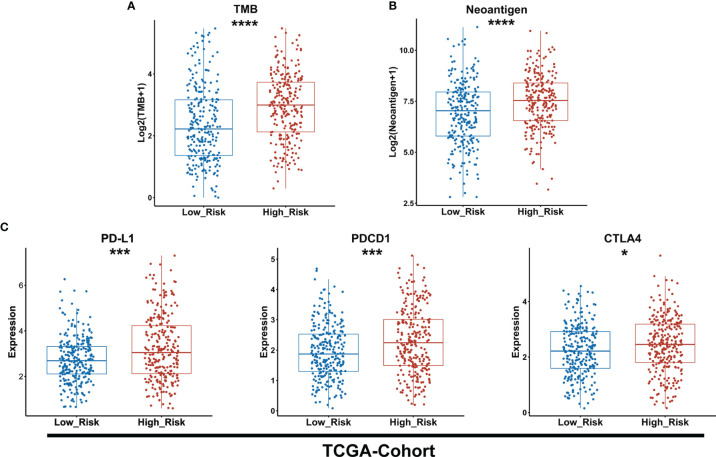
The high-risk group exhibits a higher level of TMB and neoantigen as well as PD-L1, PDCD1, and CTLA4 expression than the lower-risk group. **(A, B)** Boxplot of TMB and neoantigen between low and high-risk groups of the TCGA cohort. **(C)** Statistical analysis of the expression of PD-L1, PDCD1, CTLA4 between low- and high-risk groups in TCGA cohort. *P < 0.05; ***P < 0.001; ****P < 0.0001.

To predict the sensitivity to ICIs between low- and high-risk groups as classified by the GDPLichi model, we further examined immunotherapy-related markers such as tumor mutation burden (TMB), neoantigen, and expression of PDCD1 (PD-1), CD274 (PD-L1), and CTLA4. The degree of TMB and neoantigen in the high-risk group was significantly higher as compared to the low-risk group in the TCGA-Cohort (**Figures 6A, B**). A significantly higher expression of PD-L1, PDCD1, and CTLA4 was also observed in the high-risk group as compared to the low-risk group in the TCGA-Cohort (**Figure 6C**) as well as the three GEO validation cohorts (**Supplementary Figures S5A–C**). These results indicated that the high-risk group might be more sensitive to immunotherapy. It also suggested that the GDPLichi model may help to predict the response to ICIs of LUAD patients.

### Gene Mutation Pattern Between Low- and High-Risk Groups Classified by GDPLichi Model

**Figure 7:**
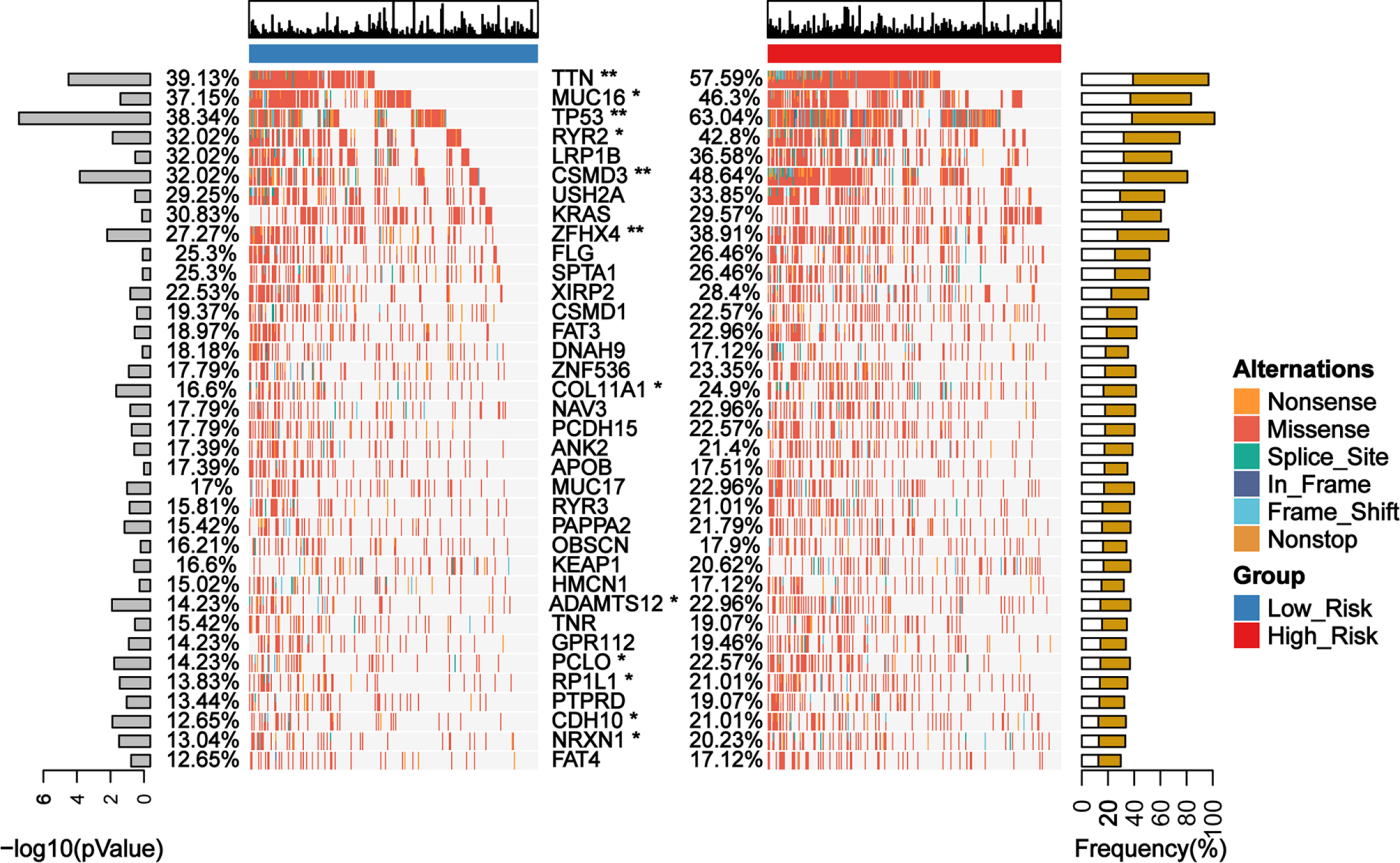
Mutation landscape of genes with significant difference between low- and high-risk subgroups in TCGA LUAD cohort. *P < 0.05; **P < 0.01.

We identified 12 candidates from the top 25 frequently mutated genes in the TCGA cohort of LUAD patients, which exhibited a significant difference between the low- and the high-risk group classified by GDPLichi (**Figure 7**). Recent studies reported that mutations in the TTN and MUC16 genes indicated high TMB (35, 36) and could be used to predict immunotherapy efficacy (37). TTN mutation status can independently predict immunotherapy prognosis in lung adenocarcinoma patients after ICIs (38). MUC16 mutation was associated with greater response rates associated with ICIs response and overall survival (39). TP53 is a well-known tumor suppressor gene with mutation occurring in more than 50 percent of all malignancies. TP53 mutation is usually associated with a poor prognosis. Some studies also reported that loss of CSMD3 results in increased proliferation of airway epithelial cells in the LUAD (40). Ovarian carcinoma patients with CSMD3 mutation had sustained responses to anti-PD1 without prior chemotherapy (41). Somatic mutations in the ZFHX4 gene are associated with poor overall survival in Chinese lung cancer patients (42). The mutation of RYR2 is a significant biomarker associated with high TMB in LUAD (43). Patients with lung adenocarcinoma with the ADAMTS12 mutation would have a worse prognosis (44). Taken together, these results suggested that the high-risk group might be more sensitive to ICIs and GDPLichi model may predict the response to ICIs.

### ICIs Response Prediction Between Low- and High-Risk Groups of LUAD Patients Classified by GDPLichi Model

**Figure 8:**
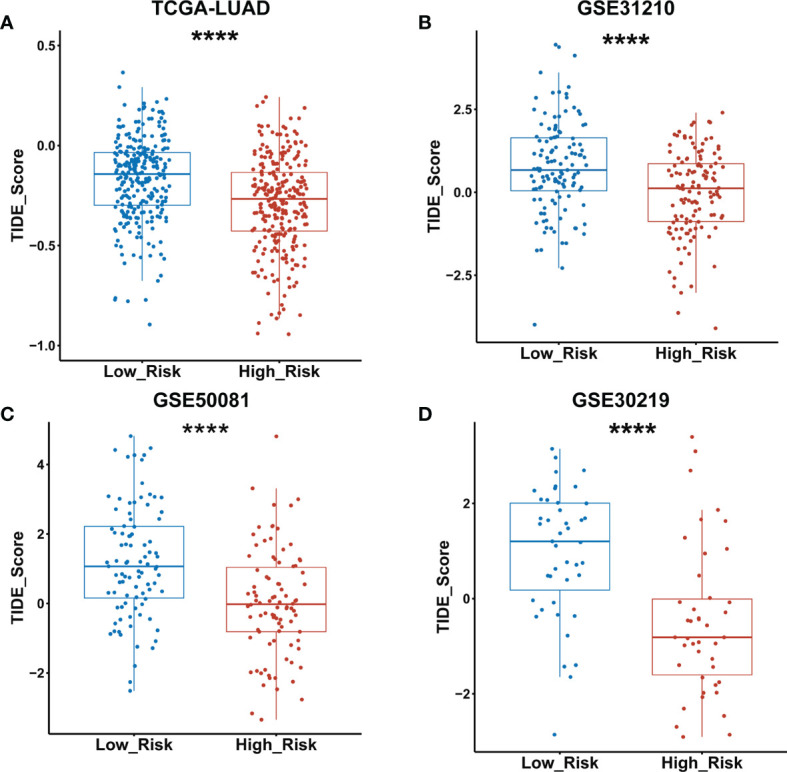
TIDE score was significantly lower in the high- as compared to the low-risk group classified by the GDPLichi model. **(A–D)** Statistical analysis of TIDE scores between low and high-risk groups divided by GDPLichi model in the TCGA LUAD cohort and the three other external validation GEO datasets. ****P < 0.0001.

The TIDE algorithm has been proved to help predict ICIs response of LUAD patients with high accuracy (22). Therefore, we calculated the TIDE scores of both low- and high-risk groups of the TCGA LUAD cohort as classified by the GDPLichi model. The results revealed that the TIDE score in the high-risk group was significantly lower than the low-risk group (**Figure 8A**). Similar results were observed in the other three external GEO datasets (**Figures 8B–D**). These results suggested that the high-risk group has a lower chance of antitumor immune escape and exhibiting a higher response rate of ICIs treatment.

## Discussion

Lung cancer ranks second in incidence and top in mortality among malignancies worldwide (1). Recently, immunotherapy has become an important new therapeutic approach in treating multiple types of cancer with promising results. It has greatly changed the landscape of cancer care. Many studies have shown that mutations in DDR pathway genes are associated with the prognosis of LUAD patients (20, 21), however, using the DDR gene expression profile as a molecular signature to predict the response to ICIs of LUAD patients has not been reported yet. In this study, we constructed a GDPLichi model based on seven DDR genes (DUT, MGMT, POLH, RAD1, RAD17, TYMS, and YWHAG), to classify LUAD into two distinct subgroups: low- and high-risk groups. Thymidylate synthase (TYMS) is a critical target for cancer chemotherapy (45). Tyrosine 3-Monooxygenase/Tryptophan 5-Monooxygenase Activation Protein Gamma (YWHAG) is also known as 14-3-3γ. A recent study reported that knockdown of YWHAG suppresses epithelial-mesenchymal transition (EMT) and reduces the metastatic potential of human NSCLC (46). O-6-Methylguanine-DNA Methyltransferase (MGMT) catalyzes the transfer of methyl groups from O(6)-alkylguanine and other methylated moieties of the DNA to its molecule. A low protein expression of MGMT was found in the bronchial epithelium of patients with lung cancer as compared to healthy controls, suggesting that there is a negative correlation between MGMT expression and lung cancer risk (47). DNA polymerase eta (POLH) is a DNA polymerase belonging to a subset of tumor suppressor proteins required for maintaining genome integrity (48). RAD1 encodes a component of a heterotrimeric cell cycle checkpoint complex, known as the 9-1-1 complex, that is activated to stop cell cycle progression in response to DNA damage or incomplete DNA replication. RAD17 is a cell cycle checkpoint gene required for cell cycle arrest and DNA damage repair in response to DNA damage. This protein recruits the RAD1-RAD9-HUS1 checkpoint protein complex onto chromatin after DNA damage and initiates DNA repair.

Gene Set Enrichment Analysis (GSEA) is a computational method that determines whether a priori defined set of genes shows statistically significant, concordant differences between two biological states. GSEA showed that cellular response to hypoxia, MAPK signaling, noncanonical NF-κB signaling, and cell cycle pathways relating to cell proliferation was significantly enriched in the high-risk group, which might account for a higher malignancy and poorer prognosis of LUAD patients in the high-risk group. Oxygen deprivation (hypoxia) is a feature of solid tumors that promotes genomic instability, enhanced aggressiveness, and metastases and is an important factor in treatment resistance and poor survival (49). The MAPK pathways converge in the amplification of key molecules that sustain cell proliferation, growth, and survival processes (50, 51). Noncanonical NF-κB signaling contains NIK phosphorylates IKK/and helps IKK/to phosphorylate p100. Mutations in various upstream regulators (TRAF2, TRAF3, cIAP1&2, CD40) lead to increased stability of NIK and subsequent activation of the noncanonical NF-kB pathway, and this mechanism of activation appears to be important for different cancer types including DLBC and lung cancer (52). The human cell cycle is a tightly regulated process with checkpoints in place to ensure genomic integrity. Recent studies have shown that CDK4 and CDK6 inhibitors can promote T cell activation (53) and reverse T cell exclusion, thus leading to a better response to ICIs (54). Taken together, this suggests that tumor cells in the high-risk group proliferated faster, leading to increased malignancy.

In addition, there were decreased B cells, CD8^+^ T cells, CD8^+^ central memory T cells, HSC, MDC, and immune scores found in the high-risk group. The CD8 T cell-dependent killing of cancer cells could produce interferon-gamma (IFN-γ) and then activate antitumor immunity (55). Myeloid dendritic cells (MDC) are crucial for the activation of antigen-specific CD8 T Cells. A recent study reported that anti-tumor effects of DCs can be reduced by a low DC count, low antigen presentation efficiency of tumor-infiltrating DCs, and a weak ability of DC to migrate into tumor (56). Many studies reported that B cell infiltration was associated with a favorable prognosis in NSCLC (57–60). Hematopoietic stem cells (HSC) are a very small group of source cells that can self-renew and generate various blood cells and immune cells. Tumor immune infiltrating cells migrate from blood to tumor tissues and play an important role in immune regulation. Lots of studies have shown that tumor immune infiltrating cells are closely related to the efficacy of ICIs and prognosis (61, 62).

Interestingly, we noticed that there were increased TMB, neoantigen, immune checkpoint molecules, and immuno-suppression genes’ expression in the high-risk group. Meanwhile, the expression of MHC II genes that express on antigen-presenting cells only in the low-risk group was significantly lower than in the high-risk group. It has been widely studied that higher TMB, neoantigen, and immune checkpoint molecules are indicators implicated in a better response to ICI treatment (8, 11, 12). Therefore, it is suggested that the high-risk group might be more sensitive to immunotherapies as compared to the low-risk group classified by the GDPLichi model.

We further examined genomic mutations in both the low- and high-risk groups and identified 12 candidates from the first top 25 mutated genes, whose mutation frequency has a significant difference between low- and high-risk groups classified by the GDPLichi model. Most of these genes are associated with TMB (35, 36), which could be used to predict the efficacy of immunotherapy.

The TIDE algorithm is a computational method that uses the expression profile of immune-related genes to predict the ICIs response. It is particularly successful in NSCLC and melanoma (22) and has exhibited a higher accuracy than PD-L1 expression level or TMB alone in predicting overall survival of patients treated with ICIs (13, 19, 20). Further analysis revealed that the TIDE scores in the high-risk group were significantly lower than the low-risk group, suggesting that patient of the high-risk group is more sensitive to response for ICIs. This conclusion was verified in the other external datasets (GSE31210, GSE50081, GSE30219).

In conclusion, we firstly identified two prognostically and clinically relevant subgroups of LUAD using the GDPLichi model which was constructed from seven DDR-risk genes. Patients from the high-risk group showed lower TIDE scores, and are thus more responsive to ICIs. The limit of this research was that it was retrospective, and results should thus be further confirmed by prospective studies.

## Data Availability Statement

The datasets presented in this study can be found in online repositories. The names of the repository/repositories and accession number(s) can be found in the article/**Supplementary Material**.

## Ethics Statement

Written informed consent was obtained from the individual(s) for the publication of any potentially identifiable images or data included in this article.

## Author Contributions

BH, YHL, and YLi designed the study. YLe and SD collected the data and performed the analysis. FY, TG, XX, YZ, LC, CFQ, NL, XZ, CL, YK, KH, and NW helped to analyze the data. YLe and SD wrote the paper. BH, TG, and XZ revised the paper. All the authors read and approved the final manuscript.

## Funding

This research was supported by the National Natural Science Foundation of China (grant number 81971470 and 32170913) and Shenzhen Science and Technology Commission Fund (program number JCYJ20190809143803732 and JCYJ20210324120602008) to BH and Chinese Postdoctoral Science Foundation (grant number 2020M683062) to NL.

## Conflict of Interest

The authors declare that the research was conducted in the absence of any commercial or financial relationships that could be construed as a potential conflict of interest.

## Publisher’s Note

All claims expressed in this article are solely those of the authors and do not necessarily represent those of their affiliated organizations, or those of the publisher, the editors and the reviewers. Any product that may be evaluated in this article, or claim that may be made by its manufacturer, is not guaranteed or endorsed by the publisher.

## Supplementary Material

Supplementary Figure S1 Detailed flowchart of this study.

Supplementary Figure S2
**(A–G)** Kaplan-Meier analysis of the genes included for GDPLichi construction (DUT, MGMT, POLH, RAD1, RAD17, TYMS, and YWHAG). **(H)** Example of calculating predicted survival rate related to .

Supplementary Figure S3
**(A)** PCA based on the expression profile of the seven risk genes according to different risk groups and **(B)** correlation between the GDPLichi and the seven risk genes in the three GEO validation cohorts.

Supplementary Figure S4
**(A)** Kaplan-Meier plots of the survival probability for low- and high-risk subgroups, **(B)** ROC curves of the GDPLichi score and seven risk genes of the classifier, **(C)** Forest plot representation of multivariate Cox model depicted association between overall survival and LUAD subgroups with other clinical factors considered in the three GEO validation cohorts.

Supplementary Figure S5
**(A–C)** Statistical analysis of the expression of PD-L1, PDCD1, CTLA4 between low- and high-risk groups in the three GEO validation cohorts

Supplementary Figure S6
**(A)** ROC curves for the performance of the GDPLichi score, TMB, PD-L1 expression, and neoantigen in predicting prognosis. **(B–E)** Correlation between the TIDE score and GDPLichi score, TMB, PD-L1 expression, and neoantigen, respectively. **(F)** ROC curves for the performance of the original seven-gene score and three-gene combination (DUT, TYMS, and YWHAG) in predicting prognosis.

Supplementary File 1 Gene list of DDR-related genes.

Supplementary File 2 Univariate Cox regression result of DDR-related genes.

Supplementary File 3 Multivariate Cox regression result of DDR-related genes.

Supplementary File 4 Jianguoyun source data download link.

Supplementary File 5 All GSEA significant pathways.

Supplementary File 6 main_code.R (source analysis R code).
