# Supplementary figures and images for: GDPLichi: a DNA Damage Repair-Related Gene Classifier for Predicting Lung Adenocarcinoma Immune Checkpoint Inhibitors Response
Source: Front Oncol. 2021 Dec 2;11:733533. doi: 10.3389/fonc.2021.733533 (PMC8713481; doi:10.3389/fonc.2021.733533)

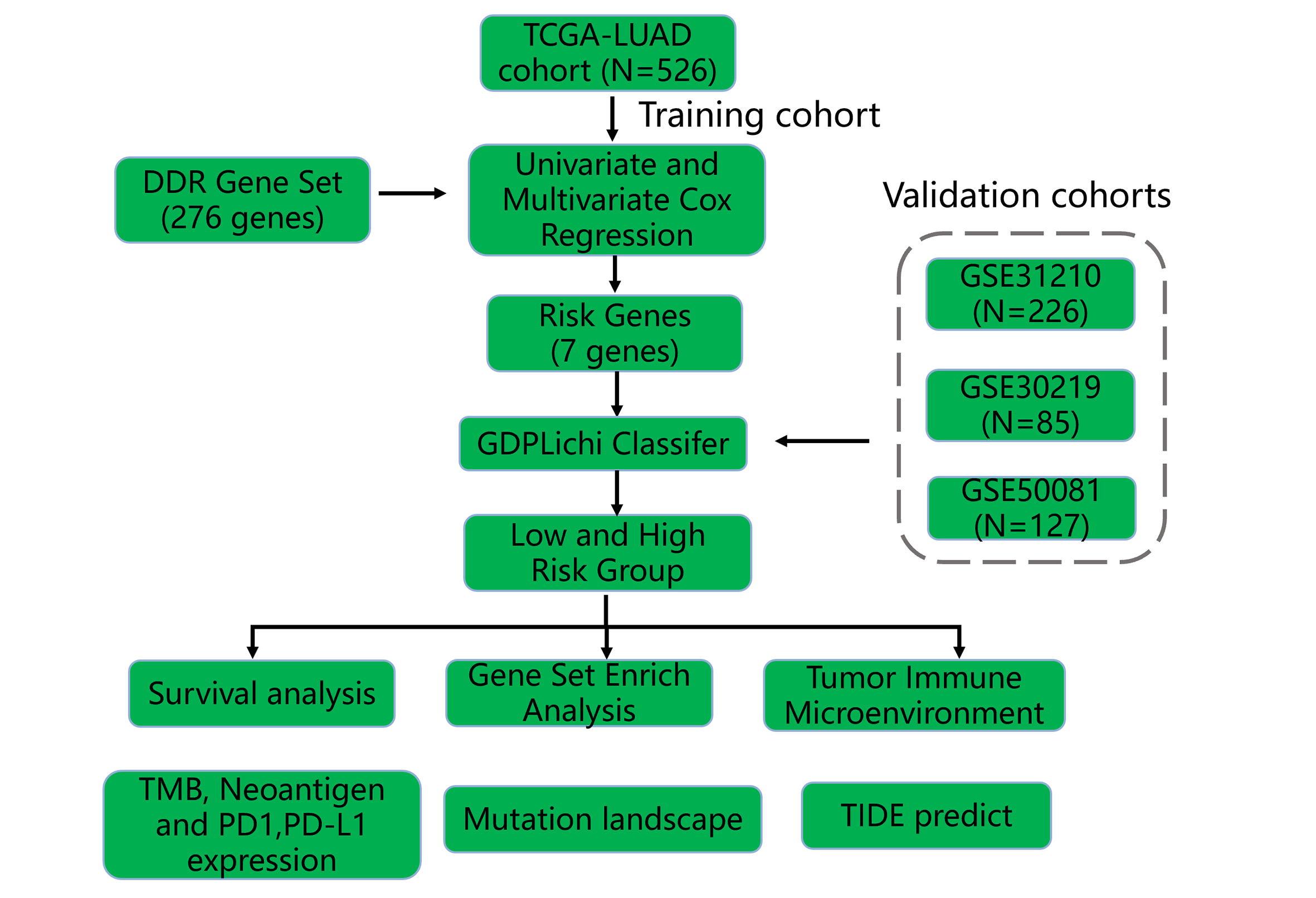

Supplement: Supplementary Figure S1 — Detailed flowchart of this study. [file Image_1.tif]

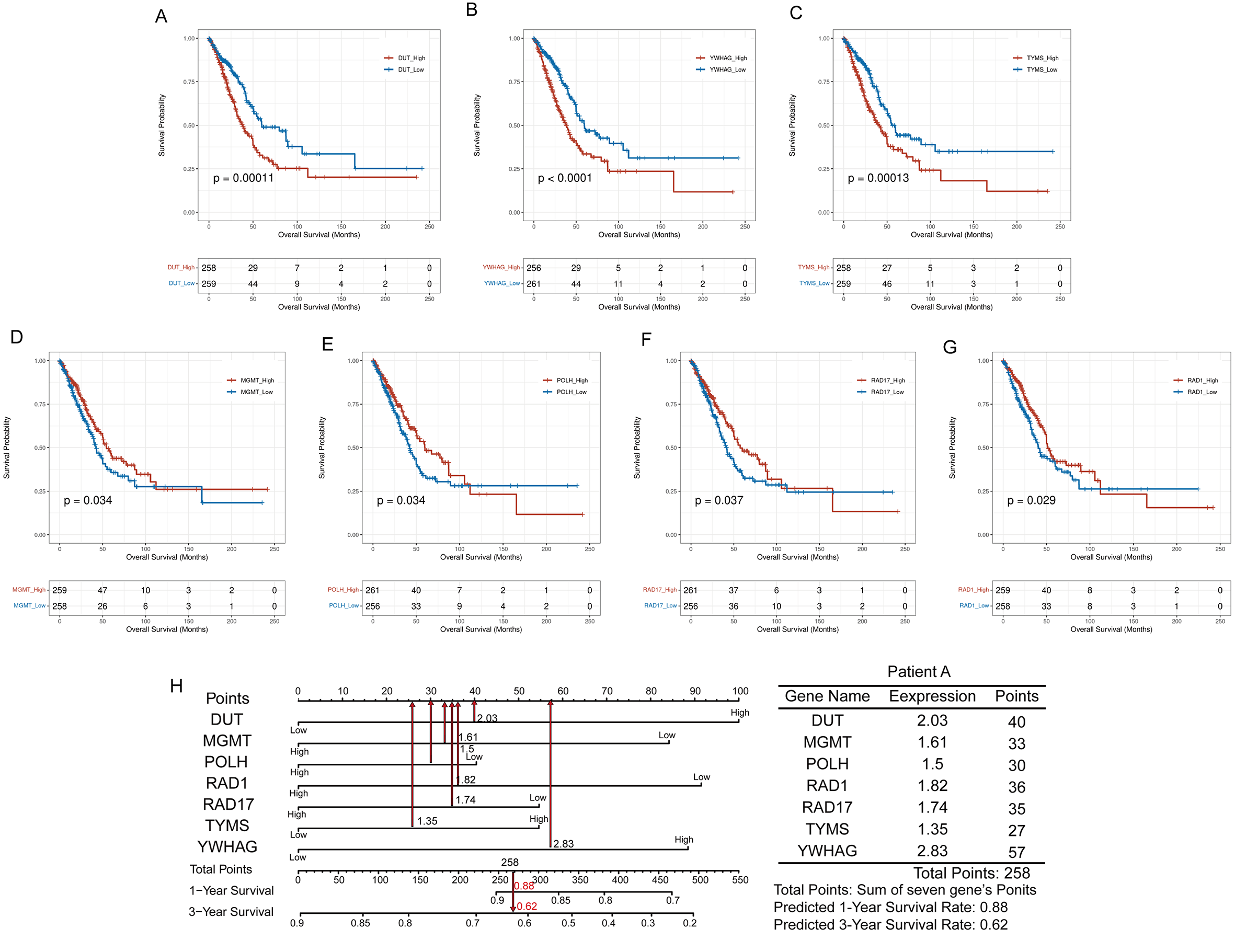

Supplement: Supplementary Figure S2 — (A–G) Kaplan-Meier analysis of the genes included for GDPLichi construction (DUT, MGMT, POLH, RAD1, RAD17, TYMS, and YWHAG). (H) Example of calculating predicted survival rate related to . [file Image_2.tif]

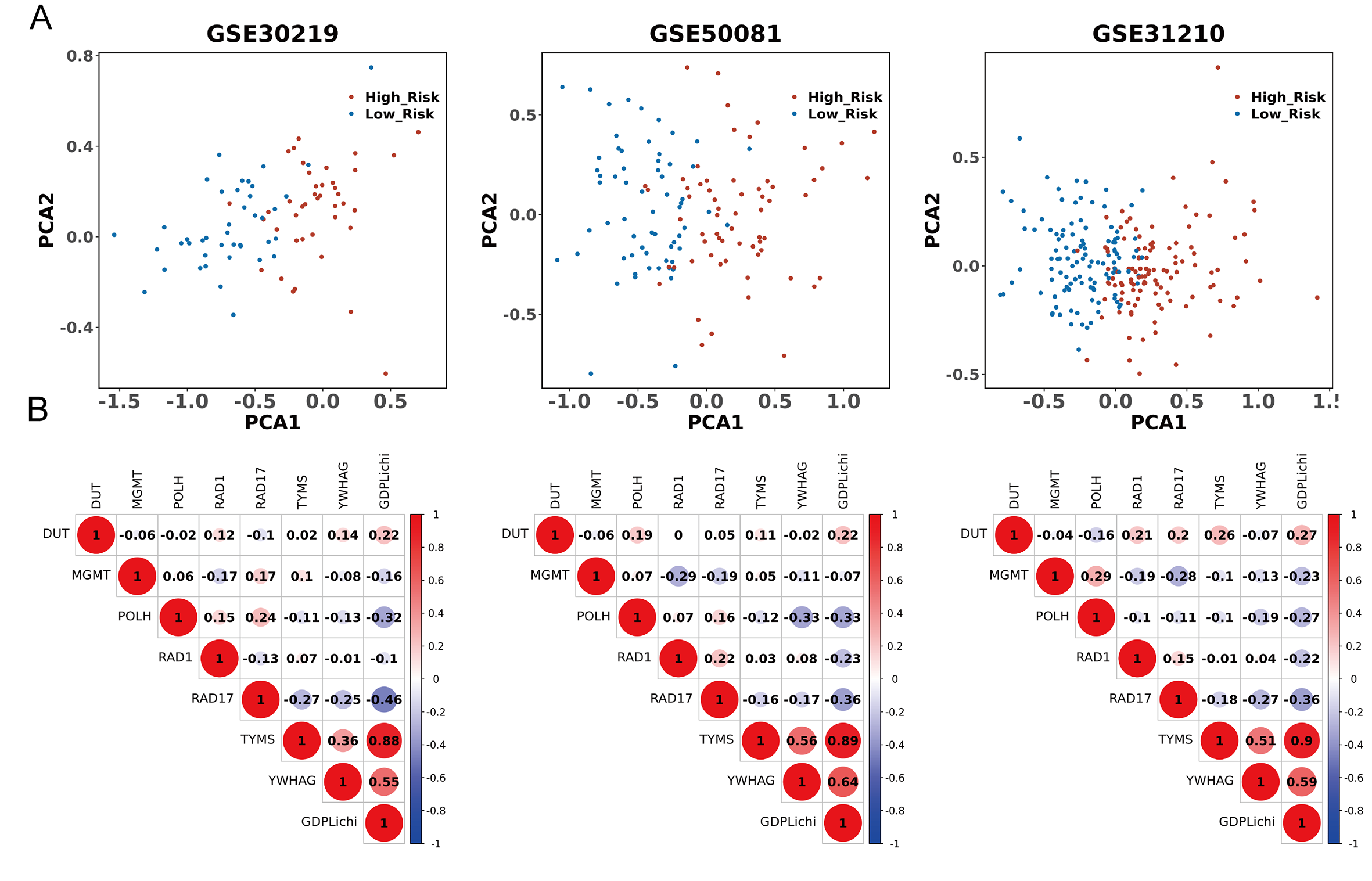

Supplement: Supplementary Figure S3 — (A) PCA based on the expression profile of the seven risk genes according to different risk groups and (B) correlation between the GDPLichi and the seven risk genes in the three GEO validation cohorts. [file Image_3.tif]

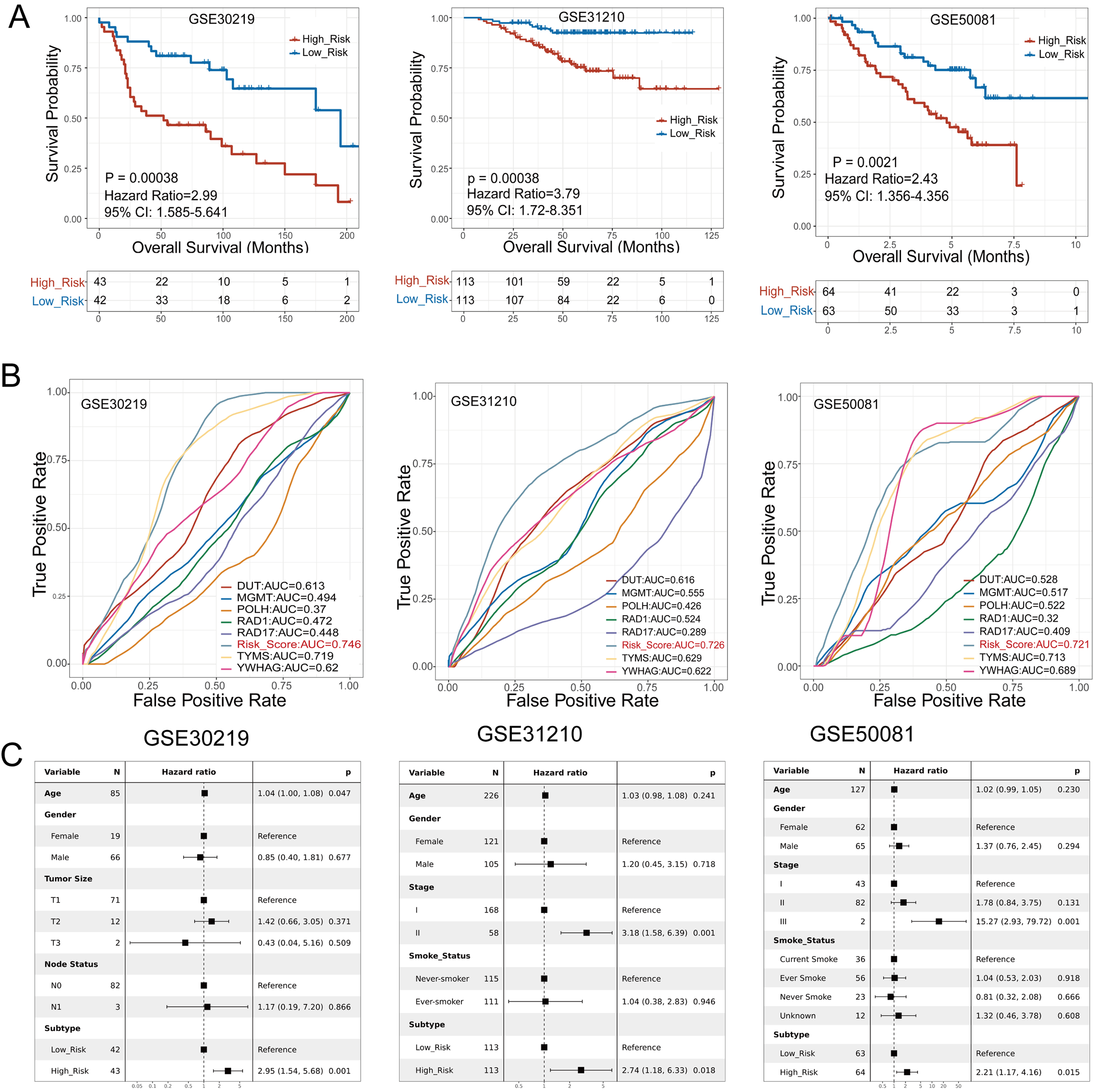

Supplement: Supplementary Figure S4 — (A) Kaplan-Meier plots of the survival probability for low- and high-risk subgroups, (B) ROC curves of the GDPLichi score and seven risk genes of the classifier, (C) Forest plot representation of multivariate Cox model depicted association between overall survival and LUAD subgroups with other clinical factors considered in the three GEO validation cohorts. [file Image_4.tif]

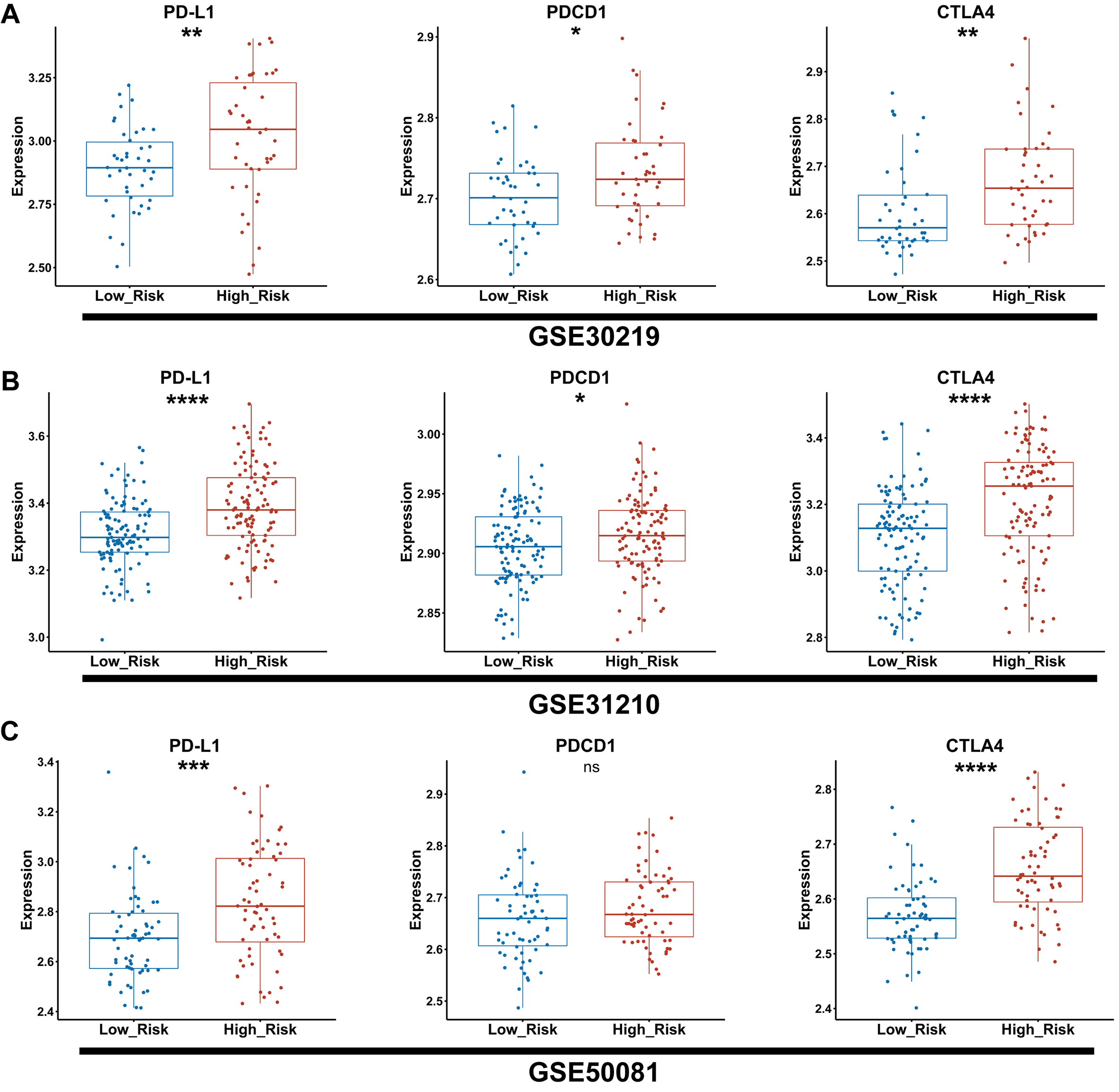

Supplement: Supplementary Figure S5 — (A–C) Statistical analysis of the expression of PD-L1, PDCD1, CTLA4 between low- and high-risk groups in the three GEO validation cohorts [file Image_5.tif]

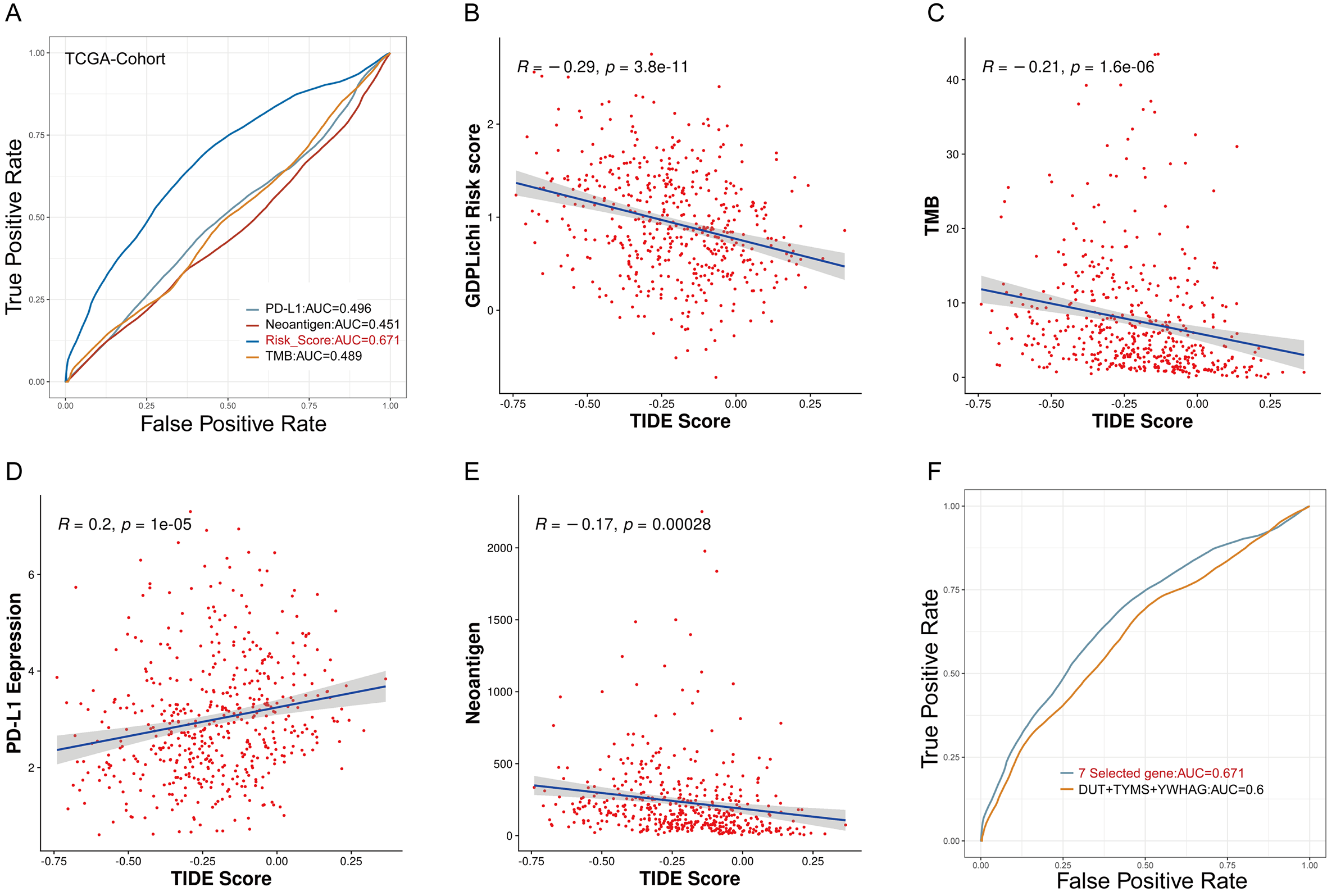

Supplement: Supplementary Figure S6 — (A) ROC curves for the performance of the GDPLichi score, TMB, PD-L1 expression, and neoantigen in predicting prognosis. (B–E) Correlation between the TIDE score and GDPLichi score, TMB, PD-L1 expression, and neoantigen, respectively. (F) ROC curves for the performance of the original seven-gene score and three-gene combination (DUT, TYMS, and YWHAG) in predicting prognosis. [file Image_6.tif]
