# Supplementary material for: GDPLichi: a DNA Damage Repair-Related Gene Classifier for Predicting Lung Adenocarcinoma Immune Checkpoint Inhibitors Response
Source: Front Oncol. 2021 Dec 2;11:733533. doi: 10.3389/fonc.2021.733533 (PMC8713481; doi:10.3389/fonc.2021.733533)
Supplement: Supplementary File 4 — Jianguoyun source data download link. [file DataSheet_4.zip › Table 3.DOCX]

**## 1 Cox regression model**

library(glmnet)

library(data.table)

library(survival)

library(rms)

library(survminer)

library(tidyverse)

UniCOX <- function(data,sur,geneList,dir,threads=4,sig=0.05) {

#@ data : expression data file, which column is sample name and row is gene

#@ sur : survival data file contains OS and OS.Time, OS is the survival staus,1 is dead

# 0 is alive, OS.time is survival time

#@ geneList : genes to run uni-cox

#@ dir : result dir

#@ threads : number of threads for data.table fread function

#@ sig : singnificance level default is 0.05

dir.create(dir,recursive = T,showWarnings = F)

data=fread(data,data.table = F,nThread = threads)

rownames(data)=data$V1

data=dplyr::select(data,-V1)

sur=read.table(sur,header = T,stringsAsFactors = F)

data2=data[geneList,]

rm(data)

data2=as.data.frame(t(data2))

data2$sample=rownames(data2)

data2=dplyr::filter(data2,sample%in%sur$sample)

data2=dplyr::left_join(data2,sur)

geneList=base::intersect(geneList,colnames(data2))

## specific for HLA-* or gene with "-" in it

sp_gene_index=grepl("-",colnames(data2))

data2_tmp1=data2[,sp_gene_index]

data2_tmp2=data2[,!sp_gene_index]

sp_gene=colnames(data2_tmp1)

sp_gene_fix=strsplit(sp_gene,"-",fixed = T)

sp_gene_fix=lapply(sp_gene_fix, function(x) paste(x[[1]],x[[2]],sep="g"))

sp_gene_fix=unlist(sp_gene_fix)

colnames(data2_tmp1)=unlist(sp_gene_fix)

data2=cbind(data2_tmp1,data2_tmp2)

rm(data2_tmp1,data2_tmp2)

geneList=geneList[!geneList%in%sp_gene]

geneList=c(geneList,sp_gene_fix)

#geneList=base::intersect(geneList,colnames(data2))

univ_formulas <- sapply(geneList,

function(x) as.formula(paste('Surv(OS.time, OS)~', x)))

univ_models <- lapply( univ_formulas, function(x) coxph(x, data = data2))

univ_results <- lapply(univ_models,

function(x){

x <- summary(x)

Likelihood.p<-signif(x$logtest["pvalue"], digits=2)

Likelihood.test<-signif(x$logtest["test"], digits=2)

wald.p<-signif(x$wald["pvalue"], digits=2)

logrank.p<-signif(x$sctest["pvalue"], digits=2)

logrank.test<-signif(x$sctest["test"], digits=2)

wald.test<-signif(x$wald["test"], digits=2)

beta<-signif(x$coef[1], digits=2);#coeficient beta

HR <-signif(x$coef[2], digits=2);#exp(beta)

HR.confint.lower <- signif(x$conf.int[,"lower .95"], 2)

HR.confint.upper <- signif(x$conf.int[,"upper .95"],2)

HR <- paste0(HR, " (", HR.confint.lower, "-", HR.confint.upper, ")")

res<-c(beta, HR, Likelihood.test,Likelihood.p,wald.test, wald.p,logrank.test,logrank.p)

names(res)<-c("BetaValue", "HR (95% CI)", "Likelihood.test","Likelihood.Pvalue","Wald.test","Wald.Pvalue","Logrank.test","Logrank.Pvalue")

return(res)

}

)

res <- t(as.data.frame(univ_results, check.names = FALSE))

res=as.data.frame(res)

res$gene=str_replace(rownames(res),"g","-")

res=dplyr::select(res,gene,everything())

res_filter=dplyr::filter(res,Logrank.Pvalue<sig)

res_file=paste0(dir,"/","Univariate_Cox.stat.csv")

res_file_filter=paste0(dir,"/","Univariate_Cox.stat.filter.csv")

write.table(res,res_file,quote = F,sep=",",row.names = F)

write.table(res_filter,res_file_filter,quote = F,sep=",",row.names = F)

return(res_filter)

}

MultiCox <- function(data,sur,geneList,dir,threads=4,sig=0.05) {

#@ data : expression data file, which column is sample name and row is gene

#@ sur : survival data file contains OS and OS.Time, OS is the survival staus,1 is dead

# 0 is alive, OS.time is survival time

#@ geneList : genes to run uni-cox

#@ dir : result dir

#@ threads : number of threads for data.table fread function,default is 4

#@ sig : singnificance level default is 0.05

dir.create(dir,recursive = T,showWarnings = F)

data=fread(data,data.table = F,nThread = threads)

rownames(data)=data$V1

data=dplyr::select(data,-V1)

sur=read.table(sur,header = T,stringsAsFactors = F)

data2=data[geneList,]

rm(data)

data2=as.data.frame(t(data2))

data2$sample=rownames(data2)

data2=dplyr::filter(data2,sample%in%sur$sample)

data2=dplyr::left_join(data2,sur)

geneList=base::intersect(geneList,colnames(data2))

## specific for HLA-* or gene with "-" in it

sp_gene_index=grepl("-",colnames(data2))

sp_gene=colnames(data2)[sp_gene_index]

if (length(sp_gene>0)) {

data2_tmp1=data2[,sp_gene_index]

data2_tmp2=data2[,!sp_gene_index]

#sp_gene=colnames(data2_tmp1)

sp_gene_fix=strsplit(sp_gene,"-",fixed = T)

sp_gene_fix=lapply(sp_gene_fix, function(x) paste(x[[1]],x[[2]],sep="g"))

sp_gene_fix=unlist(sp_gene_fix)

colnames(data2_tmp1)=sp_gene_fix

data2=cbind(data2_tmp1,data2_tmp2)

rm(data2_tmp1,data2_tmp2)

geneList=geneList[!geneList%in%sp_gene]

geneList=c(geneList,sp_gene_fix)

}

data2=dplyr::filter(data2, !is.na(OS.time))

data2=dplyr::filter(data2, !is.na(OS))

data2=dplyr::filter(data2,OS.time>0)

print(geneList)

multi_formula=as.formula(paste0('Surv(OS.time, OS)~',paste(geneList,collapse = "+")))

#print(multi_formula)

multi_model=coxph(multi_formula,data=data2)

multi_result=summary(multi_model)

res_coef<-multi_result$coefficients[,-3]

colnames(res_coef)=c("BetaValue","HR","Zscore","Pvalue")

HR.confint.lower <- signif(multi_result$conf.int[,"lower .95"],2 )

HR.confint.upper<- signif(multi_result$conf.int[,"upper .95"],2)

HR<-as.data.frame(res_coef)$HR

HR <- paste0(HR, "(",HR.confint.lower, "-", HR.confint.upper,")")

multi_res<-as.data.frame(res_coef)

multi_res$HR <-HR

multi_res_filter=dplyr::filter(multi_res,Pvalue<sig)

## calculate riskScore and use median as cutoff value to devided patients in two subgroups

## and use riskScore to predict survival (ROC)

if (nrow(multi_res_filter>=1)) {

## For nomogram

geneList=rownames(multi_res_filter)

# ## specific for HLA-* or gene with "-" in it

# sp_gene_index=grepl("-",geneList)

# sp_gene=geneList[sp_gene_index]

# if (length(sp_gene>0)) {

#

# sp_gene_fix=strsplit(sp_gene,"-",fixed = T)

# sp_gene_fix=lapply(sp_gene_fix, function(x) paste(x[[1]],x[[2]],sep="g"))

# sp_gene_fix=unlist(sp_gene_fix)

# geneList=geneList[!geneList%in%sp_gene]

# geneList=c(geneList,sp_gene_fix)

# }

#

#data3=data2[,geneList]

data2$OS.time=data2$OS.time/30

multi_filter_formula=as.formula(paste0('Surv(OS.time, OS)~',paste(geneList,collapse = "+")))

dd <<- datadist(data2)

options(datadist="dd")

coxm <- rms::cph(multi_filter_formula,data=data2,x=T,y=T,surv=T)

print(coxm)

surv <- Survival(coxm)

med <- Quantile(coxm)

surv1 <- function(x)surv(1*12,lp=x)

surv2 <- function(x)surv(1*36,lp=x)

#dd=datadist(data2)

nom <- nomogram(coxm,fun=list(surv1,surv2),lp= F,

funlabel=c('1-Year Survival','3-Year Survival'),

maxscale=100,fun.at=c('0.9','0.85','0.8','0.7','0.6','0.5','0.4','0.3','0.2','0.1'))

saveRDS(nom,paste0(dir,"/nomogram_gene.Rds"))

nomo=paste0(dir,"/Nomogram_OS.pdf")

pdf(nomo,width=6,height=6)

plot(nom,xfrac=0.2,cex.axis=0.7,cex.var=0.8)

dev.off()

print("start non pred")

m=floor(coxm$stats[1]/3)

u=c(12,36)

b=coxm$stats[1]

lapply(u,function(x){

xl<-paste("Nomogram-Predicted Probability of ",x,"-Months OS")

yl<-paste("Actual ",x,"-Months OS (proportion)")

file<-paste0(dir,"/Calibration_plot.",x,"Months.pdf")

cal<- calibrate(coxm, cmethod='KM', method="boot", u=x, m=m, B=b)

pdf(file,width=6,height=6)

par(mar=c(10,5,3,2),cex = 1.0)

plot(cal,lwd=2,lty=1,

errbar.col=c(rgb(0,118,192,maxColorValue=255)),

xlim=c(0,1),ylim=c(0,1),

xlab=xl,

ylab=yl,

cex = 0.5,

col=c(rgb(192,98,83,maxColorValue=255))

)

dev.off()

})

##

coef=data.frame(gene=rownames(multi_res_filter),BetaValue=multi_res_filter$BetaValue)

FinalGeneExp = dplyr::select(data2,all_of(coef$gene) )

myFun = function(x){crossprod(as.numeric(x),coef$BetaValue)}

riskScore = apply(FinalGeneExp,1,myFun)

risk = as.vector(ifelse(riskScore > median(riskScore), "High_Risk", "Low_Risk"))

colnames(FinalGeneExp)=str_replace(colnames(FinalGeneExp),"g","-")

dat = cbind(FinalGeneExp, riskScore=as.vector(riskScore), risk, sample=data2$sample,

OS=data2$OS,OS.time=data2$OS.time)

dat=dplyr::select(dat,all_of(c("sample","riskScore","risk","OS","OS.time")),everything())

write.table(dat,paste0(dir,"/riskScore.csv"),sep = ",",quote = F,row.names = F)

multi_res_filter$gene=str_replace(rownames(multi_res_filter),"g","-")

multi_res_filter=dplyr::select(multi_res_filter,gene,everything())

res_file_filter=paste0(dir,"/","Multivariate_Cox.stat.filter.csv")

write.table(multi_res_filter,res_file_filter,quote =FALSE,sep=",",row.names=F)

library(ROCR) #使用ROCR包绘制预测模型的ROC曲线

library(caret)

pred <- prediction(dat$riskScore, dat$OS)

perf <- performance(pred,"tpr","fpr")

pref_auc=performance(pred,"auc") # shows calculated AUC for model

AUC=as.numeric(pref_auc@y.values)

AUC=signif(AUC,digits = 2)

AUC=paste0("AUC=",AUC)

pdf(paste0(dir,"/AUC.pdf"),width = 6,height = 6)

plot(perf,colorize=FALSE, col="red") #绘制ROC曲线

lines(c(0,1),c(0,1),col = "grey", lty = 4 )

text(0.9,0.1,AUC)

dev.off()

}

multi_res$gene=str_replace(rownames(multi_res),"g","-")

multi_res=dplyr::select(multi_res,gene,everything())

res_file=paste0(dir,"/","Multivariate_Cox.stat.csv")

write.table(multi_res,res_file,quote =FALSE,sep=",",row.names=F)

}

**## 2 PCA analysis**

library(data.table)

library(tidyverse)

test=fread("/data/lengyang/tcga/01.analysis/LUAD3/TCGA-LUAD_expression_zscore.tsv",data.table = F,

nThread = 10)

test_name=test$V1

test=dplyr::select(test,-V1)

group=read.table("/data/lengyang/tcga/01.analysis/LUAD3/group.tsv",header = T,stringsAsFactors = F)

rownames(test)=test_name

genes=read.table("/data/lengyang/tcga/01.analysis/LUAD3/cox/OS_0.05_0.05/Multivariate_Cox.stat.xls")

genes=dplyr::filter(genes,Pvalue<=0.05)

genes=rownames(genes)

test=as.data.frame(t(test))

test=dplyr::select(test,all_of(genes))

library(FactoMineR)

library(factoextra)

res.pca <- PCA(test, graph = T,scale.unit = F)

res.ind=get_pca_ind(res.pca)

fviz_pca_ind(res.pca, col.ind = "coord",gradient.cols = c("#00AFBB", "#E7B800", "#FC4E07"),repel =T)

te=res.ind$coord

te=as.data.frame(te)

te=dplyr::select(te,all_of(c("Dim.1","Dim.2")))

colnames(te)=c("PCA1","PCA2")

te$Sample=rownames(te)

te=dplyr::left_join(te,group)

p=ggplot(te,aes(x=PCA1,y=PCA2,color=Score_group))+geom_point()+theme_bw()+ggtitle("TCGA-LUAD")+

scale_color_nejm()+

theme(legend.position = c(0.84,0.85),

legend.title = element_blank(),

#legend.key.size = unit(1.5,"cm"),

#legend.key.height = unit(2,"cm"),

#legend.key.width = unit(2,"cm"),

legend.text = element_text(size=14,face = "bold"),

plot.title = element_text(hjust = 0.5,size=24,face = "bold"),

#axis.line = element_line(size=1),

#axis.ticks = element_line(size = 1),

axis.title=element_text(size = 20,face="bold"),

axis.text.x=element_text(size=20,face = "bold"),

axis.text.y=element_text(size=16,face = "bold"),

panel.background = element_rect(colour = "black", size = 1.2),

panel.grid = element_blank()

)

**## 3 GDPLichi Score correlation with seven risky gene**

for (i in c("LUAD3","GSE31210","GSE50081","GSE30219")){

genes=read.table("/data/lengyang/tcga/01.analysis/LUAD3/cox/OS_0.05_0.05/Multivariate_Cox.stat.xls")

genes=dplyr::filter(genes,Pvalue<=0.05)

genes=rownames(genes)

test=fread(paste0("/data/lengyang/tcga/01.analysis/",i,"/raw.tsv"),data.table = F,nThread = 10)

test=dplyr::select(test,all_of(c(genes,"Risk_Score")))

colnames(test)=c(genes,"GDPLichi")

M=cor(test,method = "spearman")

#corrplot(M, order = "original", addCoef.col = "black",type="upper",title = "TCGA-LUAD",

# tl.col="black",col=colorRampPalette(c("blue","white","red"))(200))

dir.create(paste0("/data/lengyang/tcga/01.analysis/",i,"/cor/"),recursive = T,showWarnings = F)

pdf(paste0("/data/lengyang/tcga/01.analysis/",i,"/cor/",i,"_cor.pdf"),width = 6,height = 6)

corrplot(M, order = "original", addCoef.col = "black",type="upper",

tl.col="black",col=colorRampPalette(c("blue","white","red"))(200))

dev.off()

tiff(paste0("/data/lengyang/tcga/01.analysis/",i,"/cor/",i,"_cor.tiff"),width = 6,height = 6,

res=300,compression = "lzw",units = "in")

corrplot(M, order = "original", addCoef.col = "black",type="upper",

tl.col="black",col=colorRampPalette(c("blue","white","red"))(200))

dev.off()

}

**## 4 Overrall Survival analysis between two groups**

library(tidyverse)

library(survminer)

library(survival)

library(ggsci)

library(data.table)

library(extrafont)

font_import()

loadfonts(device="win")

read_data <- function(data,threads=4) {

data=fread(data,data.table = F,nThread =threads)

rownames(data)=data$V1

data=dplyr::select(data,-V1)

return(data)

}

windowsFonts(myFont = windowsFont("Arial"))

VaildSurvival <- function(betaValue,zscore,tcga=F,surdata,result_path) {

#betaValue=read.table("/data/lengyang/tcga/01.analysis/LUAD3/cox/OS_0.05_0.05/Multivariate_Cox.stat.xls",header = T,stringsAsFactors = F)

betaValue=read.table(betaValue,header = T,stringsAsFactors = F)

betaValue$gene=rownames(betaValue)

betaValue=dplyr::filter(betaValue,Pvalue<=0.05)

#ex=read.table("/data/lengyang/tcga/01.analysis/LUAD3/TCGA-LUAD_expression_zscore.tsv",header = T,stringsAsFactors = F,

# row.names = 1,sep = "\t",check.names = F)

ex=read_data(zscore)

ex=as.data.frame(t(ex),stringsAsFactors=F)

genes=rownames(betaValue)

data=dplyr::select(ex,all_of(genes))

if (tcga) {

data$Sample=rownames(data)

}else{

data$sample=rownames(data)

}

if (tcga) {

sample=data$Sample

new_sample=vector()

j=1

for(i in sample){

tmp=substr(i,1,nchar(i)-1)

new_sample[j]=tmp

j=j+1

}

data$sample=new_sample

}

cdata=read.table(surdata,header = T,stringsAsFactors = F,sep = "\t")

if (tcga) {

data=dplyr::left_join(data,cdata,by="sample")

}else{

data=dplyr::left_join(cdata,data,by="sample")

}

data$Risk_Score=0

for (gene in genes) {

data$Risk_Score=data$Risk_Score+betaValue[gene,"BetaValue"]*data[,gene]

}

med=median(data$Risk_Score)

data=dplyr::mutate(data,Score_group=if_else(Risk_Score>=med,"High_Risk","Low_Risk"))

#cdata=read.table("/data/lengyang/tcga/survival/LUAD_survival.txt",header = T,stringsAsFactors = F,sep = "\t")

#cdata=read.table(surdata,header = T,stringsAsFactors = F,sep = "\t")

#data=dplyr::left_join(data,cdata,by="sample")

#data$OS.time=data$OS.time/30

#data$PFI.time=data$PFI.time/30

fit1 <- survfit(Surv(OS.time,OS) ~ Score_group, # 创建生存对象

data = data) # 数据集来源

col=pal_nejm("default")(8)

num=length(unique(data$Score_group))

p=ggsurvplot(fit1, data = data,

pval = TRUE,

pval.size = 6,

ggtheme=theme_bw(),

risk.table = TRUE,

risk.table.title="",

palette = col[0:num],

legend.labs=c("High_Risk","Low_Risk"),

legend.title = "",

legend = c(0.85,0.85),

xlab = "Overall Survival (Months)", # 指定x轴标签,

ylab="Survival Probability"

)

#result_path="/data/lengyang/tcga/01.analysis/LUAD3/"

dir.create(result_path,showWarnings = F,recursive = T)

outfile1=paste0(result_path,"/OS.pdf")

pdf(outfile1,width=6,height=6)

print(p,newpage = FALSE)

dev.off()

outfile2=paste0(result_path,"/OS.tiff")

tiff(outfile2,width=6,height=6,res=300,compression = "lzw",units = "in")

print(p,newpage = FALSE)

dev.off()

if (tcga) {

fit2 <- survfit(Surv(PFI.time,PFI) ~ Score_group, # 创建生存对象

data = data) # 数据集来源

col=pal_nejm("default")(8)

num=length(unique(data$Score_group))

p2=ggsurvplot(fit2, data = data,

pval = TRUE,

pval.size = 6,

ggtheme=theme_bw(),

risk.table = TRUE,

risk.table.title="",

palette = col[0:num],

legend.labs=c("High_Risk","Low_Risk"),

legend.title = "",

legend = c(0.85,0.85),

xlab = "Progression Free Interval (Months)", # 指定x轴标签,

ylab="Survival Probability"

)

outfile1=paste0(result_path,"/PFI.pdf")

pdf(outfile1,width=6,height=6)

print(p2,newpage = FALSE)

dev.off()

outfile2=paste0(result_path,"/PFI.tiff")

tiff(outfile2,width=6,height=6,res=300,compression = "lzw",units = "in")

print(p2,newpage = FALSE)

dev.off()

}

write.table(data,paste0(result_path,"/raw.tsv"),quote = F,row.names = F,sep = "\t")

if (tcga) {

gp=dplyr::select(data,c("Sample","Score_group"))

}else{

gp=dplyr::select(data,c("sample","Score_group"))

}

write.table(gp,paste0(result_path,"/group.tsv"),quote = F,row.names = F,sep = "\t")

}

**## 5 SurvivalROC cruve**

library(data.table)

library(survivalROC)

library(ggsci)

library(cowplot)

read_data <- function(data,threads=4) {

data=fread(data,data.table = F,nThread =threads)

rownames(data)=data$V1

data=dplyr::select(data,-V1)

return(data)

}

data=read.table("raw.tsv",header = T,stringsAsFactors = F)

##tcga

data=dplyr::select(data,c("Sample","Risk_Score","OS","OS.time"))

expres=read_data("TCGA-LUAD.htseq_counts.tsv.count_allTumor.xlsTPM.xls")

betaValue=read.table("Multivariate_Cox.stat.xls",

header = T,stringsAsFactors = F)

betaValue=dplyr::filter(betaValue,Pvalue<=0.05)

genes=rownames(betaValue)

expres=as.data.frame(t(expres),stringsAsFactors=F)

gp=read.table("group.tsv",header = T,stringsAsFactors = F)

## if tcga

expres$Sample=rownames(expres)

i=length(genes)+1

genes[i]="Sample"

expres=dplyr::select(expres,all_of(genes))

cutoff=36

nobs <- NROW(data)

data=dplyr::left_join(data,expres)

#data=arrange(data, Risk_Score)

genes[i]="Risk_Score"

data1=data.frame(FP=1,TP=1,marker="tmp")

for (marker in genes) {

print(marker)

pred= survivalROC(Stime=data$OS.time,## suvival time

status=data$OS,## dead or live

marker = data[,marker], ## marker value

predict.time = cutoff,## suvival time cutoff value

span = 0.25*nobs^(-0.20))##span,NNE method namde

auc=pred$AUC

auc=round(auc,3)

data2=data.frame(FP=pred$FP,TP=pred$TP,marker=paste0(marker,":AUC=",auc))

data1=rbind(data1,data2)

}

data1=dplyr::filter(data1,marker!="tmp")

p=ggplot(data1,aes(x=FP,y=TP,color=marker))+geom_line()+scale_color_nejm()+theme_bw()+xlab("False Positive Rate")+ylab("True Positive Rate")

p

ggsave("Survival_ROC.tiff",p,width = 8,height = 6,compression="lzw")

ggsave("Survival_ROC.pdf",p,width = 8,height = 6)

write.table(data1,"Survival_ROC.tsv",quote = F,row.names = F,sep="\t")

data=dplyr::left_join(gp,data,by="Sample")

write.table(data,"Survival_ROC_raw.tsv",quote = F,row.names = F,sep="\t")

data=arrange(data, Risk_Score)

ggplot(test,aes(x=sample,y=Risk_Score,color=Score_group))+geom_point(size=0.05)+theme_bw()+

theme(

axis.title.x = element_blank(),

axis.ticks.x = element_blank(),

axis.text.x = element_blank(),

panel.grid.major=element_line(colour=NA)

)

ggsave("test.tiff",width = 8,height = 6,compression="lzw")

test$OS=as.factor(test$OS)

ggplot(test,aes(x=sample,y=OS.time,color=OS))+geom_point(size=1)+theme_cowplot()+

theme(

axis.title.x = element_blank(),

axis.ticks.x = element_blank(),

axis.text.x = element_blank(),

panel.grid.major=element_line(colour=NA)

)

**## 6 forest plot**

library(forestmodel)

library(survival)

library(dplyr)

pheno=read.table("TCGA-LUAD.GDC_phenotype.tsv",header = T,stringsAsFactors = F,sep = "\t",fill = T,quote = "")

##TCGA-LUAD.GDC_phenotype.tsv downloaded from https://xenabrowser.net/datapages/

pheno2=dplyr::select(pheno, all_of(c("submitter_id.samples","age_at_index.demographic","tumor_stage.diagnoses","gender.demographic",

"stopped_smoking_year","tobacco_smoking_history","year_of_tobacco_smoking_onset",

"number_pack_years_smoked","pack_years_smoked.exposures","years_smoked.exposures")))

#pheno2=dplyr::mutate(pheno2,somke_status=if_)

sm_status=c("Lifelong Non-smoker","Current smoker","Current reformed smoker for > 15 years","Current reformed smoker for ≤15 years","Current reformed smoker, duration not specified")

sm_value=c(1,2,3,4,5)

pheno2=dplyr::filter(pheno2,!is.na(tobacco_smoking_history))

pheno2$Smoke_Status=pheno2$tobacco_smoking_history

pheno2$Smoke_Status <- plyr::mapvalues(x = pheno2$Smoke_Status, from = sm_value, to = sm_status)

pheno2$Stage=pheno2$tumor_stage.diagnoses

#pheno2$Stage <- plyr::mapvalues(x = pheno2$Stage, from =unique(pheno2$tumor_stage.diagnoses) , to =

# c("Ⅲ"),)

pheno3=dplyr::select(pheno2,all_of(c("submitter_id.samples","age_at_index.demographic","gender.demographic",

"Stage","Smoke_Status")))

colnames(pheno3)=c("Sample","Age","Gender","Stage","Smoke_Staus")

group=read.table("/data/lengyang/tcga/01.analysis/LUAD3/group.tsv",header = T,stringsAsFactors = F)

colnames(group)=c("Sample","Subtype")

pheno3=dplyr::left_join(group,pheno3)

pheno3=dplyr::filter(pheno3,!is.na(Age))

sur2=read.table("TCGA-LUAD.survival.tsv",header = T,stringsAsFactors = F)

sur2=dplyr::select(sur2,all_of(c("sample","OS","OS.time")))

sur2=dplyr::rename(sur2,Sample=sample)

pheno3=dplyr::left_join(pheno3,sur2)

pheno4=dplyr::select(pheno3,-Sample)

pheno4=dplyr::filter(pheno4, Stage!="not reported")

pheno4=dplyr::rename(pheno4, stage=Stage)

pheno4$Stage=pheno4$stage

pheno4$Stage=plyr::mapvalues(pheno4$Stage,from = unique(pheno4$Stage),to=c("stage i","stage i","stage iii","stage iv","stage ii","stage ii",

"stage i","stage ii","stage iii"))

pheno4=dplyr::select(pheno4,-stage)

pheno4=dplyr::rename(pheno4,Smoke_Status=Smoke_Staus)

pheno4=dplyr::filter(pheno4,Smoke_Status!="Current reformed smoker, duration not specified")

pheno4$Smoke_Status=plyr::mapvalues(pheno4$Smoke_Status, from = unique(pheno4$Smoke_Status),

to=c("Reformed smoker > 15 years","Reformed smoker ≤15 years","Never smoker","Current smoker"))

pheno4=mutate(pheno4,Gender=if_else(Gender=="female","Female","Male"))

pheno4$Stage=plyr::mapvalues(pheno4$Stage,from = unique(pheno4$Stage), to=c("I","III","IV","II"))

pheno4$Subtype=factor(pheno4$Subtype,levels = c("Low_Risk","High_Risk"))

pheno4$Stage=factor(pheno4$Stage,levels = c("I","II","III","IV"))

pheno4$Smoke_Status=factor(pheno4$Smoke_Status,levels = c("Current smoker","Never smoker","Reformed smoker > 15 years",

"Reformed smoker ≤15 years"))

pheno4=dplyr::select(pheno4,all_of(c("Age","Gender","Stage","Smoke_Status","Subtype","OS","OS.time")))

coxphmodel <- coxph(Surv(OS.time, OS) ~ ., pheno4)

te=forest_model(coxphmodel,factor_separate_line = T)

te

pdf(file = "TCGA_LUAD_nomogram.pdf",height = 8.5,width =8.5)

te

dev.off()

**## 7 gene difference boxplot (CTLA4,PD-1,PD-L1, cell cycle gene, cell Response to Hypoxia;etc and TIDE in two groups)**

plotKeyGene <- function(expres,gp,gene,category,result_path,tcga=F) {

ex=read_data(data=expres)

gp=read.table(gp,header = T,stringsAsFactors = F,sep = "\t")

ex=as.data.frame(t(ex),stringsAsFactors=F)

if (tcga) {

ex$Sample=rownames(ex)

ex=dplyr::left_join(gp,ex,by="Sample")

}else{

ex$sample=rownames(ex)

ex=dplyr::left_join(gp,ex,by="sample")

}

samples=ex$Sample

gene2=gene

gene2=base::intersect(gene2,colnames(ex))

i=length(gene)+1

gene2[i]="Score_group"

ex=dplyr::select(ex,all_of(gene2))

ex=tidyr::gather(ex,gene,exp,-Score_group)

ex=dplyr::mutate(ex,logex=log2(exp+1))

ex2=ex

ex2$Score_group=factor(ex2$Score_group,levels = c("Low_Risk","High_Risk"))

ex2=arrange(ex2,Score_group)

nejm=pal_nejm("default")(8)

nejm=nejm[1:2][2:1]

p3=ggboxplot(ex2,x="gene",y="logex",color = "Score_group",palette=nejm,add = "jitter")+

stat_compare_means(aes(group = Score_group), label = "p.signif",size=6,vjust = 0.4)+

rotate_x_text(45)+xlab("")+ylab("Expression")

dir.create(paste0(result_path,"/",category),recursive = T,showWarnings = F)

outfile1=paste0(result_path,"/",category,"/",category,".tiff")

outfile2=paste0(result_path,"/",category,"/",category,".pdf")

ggsave(outfile1,p3,width = 2*length(gene2),height =6 ,compression = "lzw")

ggsave(outfile2,p3,width = 2*length(gene2),height =6)

p4=ggboxplot(ex2,x="gene",y="logex",color = "Score_group",palette=nejm,add = "jitter")+

stat_compare_means(aes(group = Score_group), label = "p.signif",size=6,vjust = 0.8)+

xlab("")+ylab("Expression")+

facet_wrap(~gene, scales="free", ncol=4)+theme_bw()

outfile3=paste0(result_path,"/",category,"/",category,"_facet.tiff")

outfile4=paste0(result_path,"/",category,"/",category,"_facet.pdf")

if (length(gene2)<4) {

ggsave(outfile3,p4,width =11/4*length(gene2) ,height =11/4 ,compression = "lzw")

ggsave(outfile4,p4,width=11/4*length(gene2) ,height =11/4)

}else{

ggsave(outfile3,p4,width =11 ,height =ceiling(length(gene2)/4)*11/4 ,compression = "lzw")

ggsave(outfile4,p4,width=11 ,height =ceiling(length(gene2)/4)*11/4)

}

for (ge in gene) {

ex3=dplyr::filter(ex,gene==ge)

p5=ggboxplot(ex3,x="Score_group",y="logex",color="Score_group",palette=nejm,add = "jitter",order = c("Low_Risk","High_Risk"))+labs(title = ge)+

stat_compare_means(aes(group = Score_group), label = "p.signif",size=6,label.x.npc="center")+xlab("")+ylab("Expression")+

theme(legend.position = "none",

plot.title = element_text(hjust = 0.5,size=24,face = "bold"),

axis.line = element_line(size=1),

axis.ticks = element_line(size = 1),

axis.title=element_text(size = 20,face="bold"),

axis.text.x=element_text(size=20,face = "bold"),

axis.text.y=element_text(size=16,face = "bold")

)

outfile5=paste0(result_path,"/",category,"/",ge,".tiff")

outfile6=paste0(result_path,"/",category,"/",ge,".pdf")

ggsave(outfile5,p5,width = 6,height =6 ,compression = "lzw")

ggsave(outfile6,p5,width = 6,height =6)

}

}

**## 8 different immune cell boxplot**

PlotImmuneCell=function(immu,gp,category,result_path,tcga=F){

data=fread(immu,data.table = F,nThread = 4)

row_name=data$cell_type

data=dplyr::select(data,-cell_type)

rownames(data)=row_name

data=as.data.frame(t(data),stringsAsFactors = F)

if (category=="mcp_counter") {

data=dplyr::rename(data,"Macrophage"="Macrophage/Monocyte")

}

data$sample=rownames(data)

gp=read.table(gp,header = T,stringsAsFactors = F)

if (tcga) {

gp=dplyr::rename(gp,sample=Sample)

}

data=dplyr::left_join(gp,data,by="sample")

data=dplyr::select(data,-sample)

data=tidyr::gather(data,cell,proportion,-Score_group)

data$Score_group=factor(data$Score_group,levels = c("Low_Risk","High_Risk"))

p6=ggboxplot(data,x="cell",y="proportion",color = "Score_group",palette=nejm,add = "jitter",oorder = c("Low_Risk","High_Risk"))+

stat_compare_means(aes(group = Score_group), label = "p.signif",size=8,vjust = 0.8)+

xlab("")+

facet_wrap(~cell, scales="free", ncol=4)+theme_bw()

dir.create(paste0(result_path,"/",category),showWarnings = F,recursive = T)

outfile7=paste0(result_path,"/",category,"/immune_facet.tiff")

outfile8=paste0(result_path,"/",category,"/immune_facet.pdf")

if (length(unique(data$cell))<4) {

ggsave(outfile7,p6,width =11/4*length(unique(data$cell)) ,height =11/4 ,compression = "lzw")

ggsave(outfile8,p6,width=11/4*length(unique(data$cell)) ,height =11/4)

}else{

ggsave(outfile7,p6,width =11 ,height =ceiling(length(unique(data$cell))/4)*11/4 ,compression = "lzw")

ggsave(outfile8,p6,width=11 ,height =ceiling(length(unique(data$cell))/4)*11/4)

}

for (cel in unique(data$cell)) {

data1=dplyr::filter(data,cell==cel)

p7=ggboxplot(data1,x="Score_group",y="proportion",color="Score_group",palette=nejm,add = "jitter")+labs(title = cel)+

stat_compare_means(aes(group = Score_group), label = "p.signif",size=6,label.x.npc="center")+xlab("")+

theme(legend.position = "none",

plot.title = element_text(hjust = 0.5,size=24,face = "bold"),

axis.line = element_line(size=1),

axis.ticks = element_line(size = 1),

axis.title=element_text(size = 20,face="bold"),

axis.text.x=element_text(size=20,face = "bold"),

axis.text.y=element_text(size=16,face = "bold")

)

outfile9=paste0(result_path,"/",category,"/",cel,".tiff")

outfile10=paste0(result_path,"/",category,"/",cel,".pdf")

ggsave(outfile9,p7,width = 6,height =6 ,compression = "lzw")

ggsave(outfile10,p7,width = 6,height =6)

}

}

## 9 GSEA analysis

library(clusterProfiler)

data=read_data(data="TCGA-LUAD.htseq_counts.tsv.count_allTumor.xlsTPM.xls")

gp=read.table("group.tsv",header = T,stringsAsFactors = F)

data=as.data.frame(t(data),stringsAsFactors=F)

data$Sample=rownames(data)

data=dplyr::left_join(gp,data)

data=dplyr::select(data,-Sample)

data=tidyr::gather(data,gene,ex,-Score_group)

data=data%>%group_by(gene,Score_group)%>%summarise_all(mean)

data=spread(data,Score_group,ex)

data=dplyr::filter(data,High_Risk>0)

data=dplyr::filter(data,Low_Risk>0)

data=mutate(data,fc=High_Risk/Low_Risk)

data=dplyr::filter(data,!is.nan(fc))

data=dplyr::filter(data,!is.(fc))

keygene=bitr(data$gene,fromType = "SYMBOL",toType = "ENTREZID",OrgDb = org.Hs.eg.db)

keygene=dplyr::rename(keygene,gene=SYMBOL)

data=dplyr::left_join(keygene,data)

genelist=data$fc

names(genelist)=data$ENTREZID

genelist <- sort(genelist, decreasing = TRUE)

library(ReactomePA)

res=gsePathway(geneList = genelist)

res=dplyr::filter(res@result, p.adjust<=0.05&abs(NES)>1)
