# Supplementary material for: GDPLichi: a DNA Damage Repair-Related Gene Classifier for Predicting Lung Adenocarcinoma Immune Checkpoint Inhibitors Response
Source: Front Oncol. 2021 Dec 2;11:733533. doi: 10.3389/fonc.2021.733533 (PMC8713481; doi:10.3389/fonc.2021.733533)
Supplement: Supplementary File 5 — All GSEA significant pathways. [file Table_1.docx]

**Raw data jianguoyun download link**

1. **TCGA cohort**

**TCGA LUAD gene expression file:** https://www.jianguoyun.com/p/DQGsgKEQyP6sBxjejv8D

**TCGA LUAD Survival data file:** https://www.jianguoyun.com/p/DZwyanYQyP6sBxjkjv8D

**TCGA LUAD Phenotype file:** https://www.jianguoyun.com/p/Db4E-2cQyP6sBxj9jv8D

**TCGA LUAD mutation file:** https://www.jianguoyun.com/p/DQgTI7YQyP6sBxiFj_8D

**TCGA LUAD Neoantigen file:** https://www.jianguoyun.com/p/Df40aIUQyP6sBxiKj_8D

**TCGA LUAD TMB file:** https://www.jianguoyun.com/p/DSWddB8QyP6sBxicj_8D

**TCGA LUAD Xcell file :** https://www.jianguoyun.com/p/DWZRW8sQyP6sBxifj_8D

1. **GSE31210**

**GSE31210 gene expression file:** https://www.jianguoyun.com/p/DQuh-8kQyP6sBxjZj_8D

**GSE31210 Survival data file:** https://www.jianguoyun.com/p/DR1p3cwQyP6sBxjhj_8D

**GSE31210 Phenotype file:** https://www.jianguoyun.com/p/DeUktPwQyP6sBxjmj_8D

1. **GSE31209**

**GSE31209 gene expression file:** https://www.jianguoyun.com/p/DWjmAJEQyP6sBxisj_8D

**GSE31209 Survival data file:** https://www.jianguoyun.com/p/DRMSFacQyP6sBxi-j_8D

**GSE31209 Phenotype file:** https://www.jianguoyun.com/p/DYfCCnsQyP6sBxjTj_8D

1. **GSE50081**

**GSE50081 gene expression file:** https://www.jianguoyun.com/p/DYLz-hIQyP6sBxjrj_8D

**GSE50081 Survival data file:** https://www.jianguoyun.com/p/DfIQud0QyP6sBxjuj_8D

**GSE50081 Phenotype file:** https://www.jianguoyun.com/p/DaHXNVkQyP6sBxjwj_8D
