# Supplementary material for: GDPLichi: a DNA Damage Repair-Related Gene Classifier for Predicting Lung Adenocarcinoma Immune Checkpoint Inhibitors Response
Source: Front Oncol. 2021 Dec 2;11:733533. doi: 10.3389/fonc.2021.733533 (PMC8713481; doi:10.3389/fonc.2021.733533)
Supplement: Supplementary File 6 — main_code.R (source analysis R code). [file Table_2.docx]

APLF

APTX

ASCC3

DNTT

LIG1

LIG3

LIG4

MRE11A

NBN

NHEJ1

PARG

PARP1

PARP3

PARPBP

PNKP

POLB

POLL

POLM

PRKDC

RAD50

RNF168

RNF8

TP53BP1

XRCC1

XRCC2

XRCC3

XRCC4

XRCC5

XRCC6

UBE2A

EXO1

HMGB1

MLH1

MLH3

MSH2

MSH3

MSH6

PCNA

PMS1

PMS2

POLD1

POLD2

POLD3

POLD4

RFC1

RFC2

RFC3

RFC4

RFC5

RPA1

RPA2

RPA3

RPA4

ALKBH1

ALKBH2

ALKBH3

APEX1

APEX2

APITD1

ATM

ATR

ATRIP

ATRX

BARD1

BLM

BRCA1

BRCA2

BRE

BRIP1

CCNH

CDK7

CETN2

CHAF1A

CHEK1

CHEK2

CLK2

CUL3

CUL4A

CUL5

DCLRE1A

DCLRE1B

DCLRE1C

DDB1

DDB2

DMC1

DNA2

DUT

EID3

EME1

EME2

ERCC1

ERCC2

ERCC3

ERCC4

ERCC5

ERCC6

ERCC8

FAAP100

FAAP24

FAAP20

FAM175A

FAN1

FANCA

FANCB

FANCC

FANCD2

FANCE

FANCF

FANCG

FANCI

FANCL

FANCM

FEN1

GADD45A

GADD45G

GEN1

GTF2H1

GTF2H2

GTF2H3

GTF2H4

GTF2H5

H2AFX

HELQ

HES1

HFM1

HLTF

HMGB2

HUS1

INO80

KAT5

MAD2L2

MBD4

MDC1

MGMT

MMS19

MNAT1

MPG

MPLKIP

MRPL40

MUS81

MUTYH

NABP2

NEIL1

NEIL2

NEIL3

NFATC2IP

NSMCE1

NSMCE2

NSMCE3

NSMCE4A

NTHL1

NUDT1

NUDT15

NUDT18

RRM1

RRM2

OGG1

PALB2

PARP2

PARP4

PAXIP1

PER1

POLA1

POLE

POLE2

POLE3

POLE4

POLG

POLH

POLI

POLK

POLN

POLQ

PPP4C

PPP4R1

PPP4R2

PPP4R4

PRPF19

RAD1

RAD17

RAD18

RAD23A

RAD23B

RAD51

RAD51B

RAD51C

RAD51D

RAD52

RAD54B

RAD54L

RAD9A

RBBP8

RBX1

RDM1

RECQL

RECQL4

RECQL5

REV1

REV3L

RIF1

RMI1

RMI2

RNMT

RRM2B

RTEL1

SETMAR

SHFM1

SHPRH

SLX1A

SLX1B

SLX4

SMARCAD1

SMC5

SMC6

SMUG1

SPO11

STRA13

SWSAP1

TCEA1

TCEB1

TCEB2

TCEB3

TDG

TDP1

TELO2

TOP3A

TOP3B

TOPBP1

TP53

TREX1

TREX2

TYMS

UBE2B

UBE2N

UBE2T

UBE2V2

UIMC1

UNG

USP1

UVSSA

WDR48

WRN

XAB2

XPA

XPC

ZSWIM7

PTEN

TDP2

ENDOV

SPRTN

RNF4

SMARCA4

IDH1

SOX4

WEE1

RAD9B

AEN

PLK3

EXO5

CDC5L

BCAS2

PLRG1

YWHAB

YWHAG

YWHAE

CDC25A

CDC25B

CDC25C

BABAM1

BRCC3

TTK

SMARCC1

SWI5

MORF4L1

RNF169

HERC2
